# Priming cardiovascular exercise improves complex motor skill learning by affecting the trajectory of learning-related brain plasticity

**DOI:** 10.1038/s41598-022-05145-7

**Published:** 2022-01-21

**Authors:** Nico Lehmann, Arno Villringer, Marco Taubert

**Affiliations:** 1 Department of Neurology, Max Planck Institute for Human Cognitive and Brain Sciences, Stephanstraße 1a, 04103 Leipzig, Germany; 2 Faculty of Humanities, Institute III, Department of Sport Science, Otto von Guericke University, Zschokkestraße 32, 39104 Magdeburg, Germany; 3 Mind and Brain Institute, Charité and Humboldt University, Luisenstraße 56, 10117 Berlin, Germany; 4 Center for Behavioral and Brain Science (CBBS), Otto von Guericke University, Universitätsplatz 2, 39106 Magdeburg, Germany

**Keywords:** Neuroscience, Cognitive neuroscience, Learning and memory

## Abstract

In recent years, mounting evidence from animal models and studies in humans has accumulated for the role of cardiovascular exercise (CE) in improving motor performance and learning. Both CE and motor learning may induce highly dynamic structural and functional brain changes, but how both processes interact to boost learning is presently unclear. Here, we hypothesized that subjects receiving CE would show a different pattern of learning-related brain plasticity compared to non-CE controls, which in turn associates with improved motor learning. To address this issue, we paired CE and motor learning sequentially in a randomized controlled trial with healthy human participants. Specifically, we compared the effects of a 2-week CE intervention against a non-CE control group on subsequent learning of a challenging dynamic balancing task (DBT) over 6 consecutive weeks. Structural and functional MRI measurements were conducted at regular 2-week time intervals to investigate dynamic brain changes during the experiment. The trajectory of learning-related changes in white matter microstructure beneath parieto-occipital and primary sensorimotor areas of the right hemisphere differed between the CE vs. non-CE groups, and these changes correlated with improved learning of the CE group. While group differences in sensorimotor white matter were already present immediately after CE and persisted during DBT learning, parieto-occipital effects gradually emerged during motor learning. Finally, we found that spontaneous neural activity at rest in gray matter spatially adjacent to white matter findings was also altered, therefore indicating a meaningful link between structural and functional plasticity. Collectively, these findings may lead to a better understanding of the neural mechanisms mediating the CE-learning link within the brain.

## Introduction

Mounting evidence shows that cardiovascular exercise (CE) facilitates neuromotor function and adaptive plasticity in the brain’s motor circuitry^1,2^. In the healthy brain, CE may aid to maximize motor potential in terms of skill acquisition and retention. For example, at the behavioral level, acute bouts of CE are robustly related to improved motor memory consolidation^3,4^, whereas CE interventions lasting several days or weeks associate with steeper learning curves in rodents^5^ and humans^6^. In the elderly, regular engagement in CE promotes the maintenance of motor functions and is thus an important factor supporting healthy aging^7,8^. Likewise, CE has also been shown to contribute to improved neuroprotection and rehabilitation outcomes in several neurological disorders like Parkinson’s^9^ or multiple sclerosis^10,11^, to name but a few.

Not surprisingly, recent years have witnessed an increased interest in the neurobiological phenomena through which CE improves motor functions. Understanding CE-mediated functional and structural brain changes and their behavioral consequences is important to identify relevant central nervous system biomarkers that may help to guide the optimization of CE parameters^12,13^, and of efficient CE-mimicking treatments as well^14–16^. A proposed mechanistic explanation for the seemingly broad transfer of CE training on motor functions, sometimes referred to as “motor priming”^17^, is that CE (“task A”) induces plasticity within neural circuits that are directly relevant for the acquisition or performance of another behavior (“task B”)^1,12,18,19^. For example, short-term plasticity studies in humans indicate that an increase of certain humoral parameters like lactate and BDNF^20^, altered cortical excitability^21,22^, downregulation of GABA-inhibition (short-interval intracortical inhibition) in M1^23^ or decreased movement-related beta desynchronization^24^ correlate with behavioral measures of motor learning. Although there is an ongoing debate surrounding the question of “optimal” exercise regimens in terms of neuroplasticity, previous studies in animals and humans seem to indicate that CE interventions of comparably short duration (i.e., days to weeks) and sufficiently intense to strain the anaerobic-lactic energy system are efficacious to trigger changes in neural activity, plasticity-related genes, neurotrophins, and neuronal and non-neuronal tissue structure^1,2,25^.

A by and large neglected area in the research on CE-induced transfer mechanisms on motor functions is the use of natural multi-articular movements as endpoints, i.e. of tasks involving the body axis and a comparably large range of motion in several joints (multiple degrees‐of‐freedom)^26,27^. Mastering of tasks with high coordinative complexity typically requires several sessions of practice^26,27^, and the learning process itself is accompanied by changes of the brain’s functional and structural network architecture^28–31^. The few studies aiming at improving learning of complex motor skills with acute CE showed promising results especially in neurologically impaired subjects^32,33^, but this effect was markedly less pronounced in healthy individuals^34^. Therefore, alternative CE protocols like training over longer time periods before motor skill learning might be an interesting alternative to acute CE^1^.

We recently reported the results of a randomized controlled trial where we evaluated the effects of two weeks of CE against an inactive control condition on subsequent learning of a well-established dynamic balancing task^6^. We found that subjects receiving CE showed a superior learning rate compared to controls. Furthermore, CE-induced increases in cerebral blood flow in frontal brain regions and changes in white matter microstructure in frontotemporal fiber tracts mediated the effect of CE on motor learning. While this study demonstrated the transfer potential of CE-induced brain plasticity, we were not able to address the question whether priming CE did also affect the dynamics of learning-dependent neuroplasticity. In this regard, Kleim and Jones outlined the intriguing hypothesis that CE might induce “plasticity within one set of neural circuits to promote concurrent or subsequent plasticity”^12^. This possibility is especially interesting with respect to the DBT paradigm, for it has been shown that functional and structural properties of the brain and their practice-induced reorganization covary with DBT performance and learning^30,31,35,36^.

**Figure 1:**
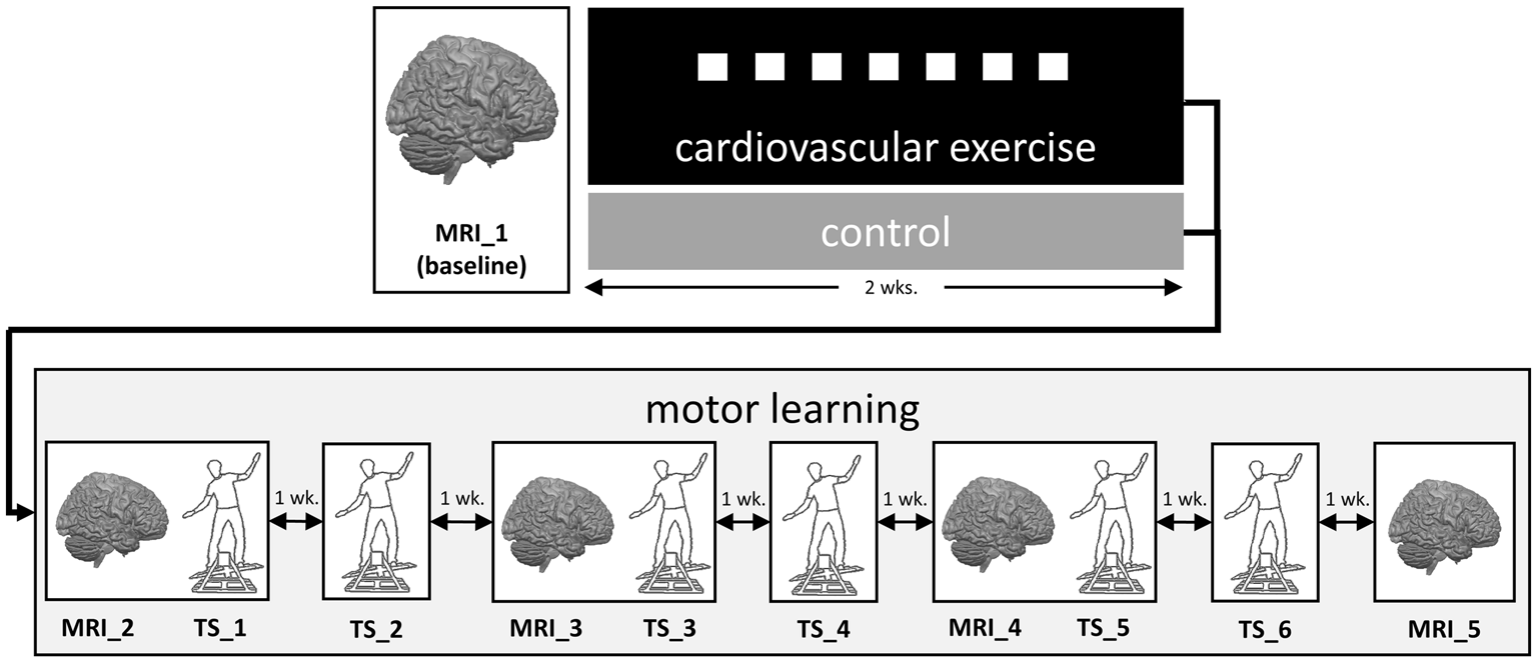
Overview of the experimental design. Subjects were randomly assigned to either 2 weeks of cardiovascular exercise (CE) or no exercise (life as usual)^6^. White squares depict seven training sessions subjects in the CE group engaged in. After the intervention period, both groups learned a complex dynamic balancing task (DBT) over six training sessions (TS) separated by 1 week, respectively. MRI measurements to assess CE- and DBT-related neuroplasticity were conducted before and at regular time intervals during the study^6,30,31,35^. *MRI* MRI measurement [number], *TS* DBT training session [number].

Extending our previous work^6^ we here focus on tracing behaviorally-relevant functional and structural plasticity during 6 weeks of DBT training^30,31,35^ that follows after a 2-week CE intervention using multimodal MRI (see Fig. 1, for experimental design). We hypothesize that subjects receiving CE show a different pattern of brain plasticity compared to controls, which in turn associates with improved DBT learning in the former group. In principle, there are two not mutually exclusive scenarios by which CE-mediated mechanisms might affect subsequent learning. Based on the assumption that structural constraints imposed by the brain determine the brain’s current range of functioning^19,36,37^, CE might build up a “structural repertoire” in the gray and/or white matter that extends the momentarily achievable performance potential of the individual. Possibly, this scenario is accompanied by between-group differences in the trajectory of functional activity changes during learning^19,38^. For instance, it has been suggested that adaptive and maladaptive plasticity in structural brain networks directly influences functional spontaneous brain activity^39^. Alternatively, CE might trigger processes that are not necessarily observable with MRI post-CE (e.g., signalling and release of growth factors like BDNF^40–43^ or VEGF^44^), nevertheless manifesting themselves in altered temporal dynamics of experience-induced brain plasticity later on^12,18^. By tracing functional and structural brain changes during DBT learning relative to the brain’s state prior to CE, we are able to determine which of these mechanistic hypotheses better conforms with the observed data.

## Results

### Sample characteristics and analysis of behavioral data

In the following, we recapitulate the most relevant sample characteristics and behavioral results that were already published in Ref.^6^. Please also see the online Supplementary Data file containing demographic, anthropometric, and aerobic fitness data of the sample. In brief, no significant between-group differences regarding demographic, anthropometric and aerobic fitness variables were detected before the experiment started. Likewise, we found that groups were comparable in terms of their extra-study physical activities (as assessed with the IPAQ-SF^45^) as well as regarding their performance in three standardized posturographic tests (see Ref.^6^, Table 1).

In our previous paper, we also reported that DBT performance significantly increased from the first to the last training session in both groups. Baseline DBT performance did not differ between groups, whereas a significantly steeper slope of the learning curve (adjusted for initial DBT performance, age, and sex) was observed for the group receiving CE^6^. Importantly, there was no significant group-by-time interaction with respect to general standing balance as assessed with posturography, suggesting that the CE intervention specifically targeted the neural mechanisms of learning the DBT^6^. Neither the temporal progression of performance within single training sessions (online learning^46^) nor motor skill consolidation^47,48^ differed between groups (Supplementary Tables 1 and 2).

### White matter plasticity during learning mediates motor learning differences between CE and controls

**Figure 2:**
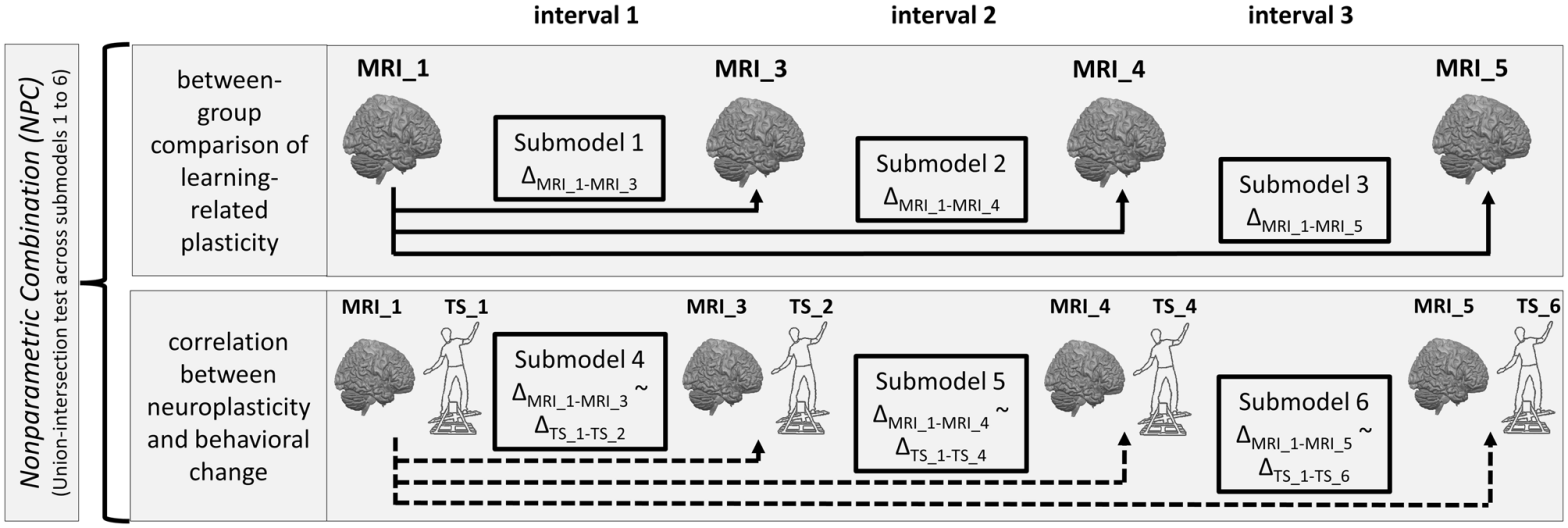
Overview of the statistical analyses using the NPC framework. For each imaging modality, we first calculated change images between baseline (MRI_1) and the MRI measurements during learning (MRI_3, MRI_4, MRI_5). Next, we set up three statistical submodels comparing groups regarding learning-related plasticity at three distinct time intervals under examination (top row). Likewise, three submodels addressing the correlation between brain changes and concurrent DBT performance changes were set up (bottom row). Union-intersection tests (UIT) were then carried out to identify clusters of voxels in which learning-related plasticity covaries with both treatment (CE vs. control) and outcome (change in DBT performance from baseline). To this end, UIT outputs a single measurement that summarizes evidence over all six submodels in every voxel.

In our previous study, CE-induced functional and structural plasticity predicted the between-group differences in motor learning^6^. However, the relevant plasticity processes occurring *during* the learning phase remained unclear. To address this issue, we calculated change images between the pre-intervention baseline MRI measurement (MRI_1) and the MRI measurements during motor learning (MRI_3, MRI_4, MRI_5), separately for each imaging modality (cf. Fig. 1). This step resulted in three images per modality signifying change from baseline that were further analyzed using the nonparametric combination (NPC) framework, which combines multiple pieces of evidence collected on the same experimental units to yield a style of meta-analytic result^49–51^. Specifically, across all intervals of the learning phase, we aimed to identify clusters of voxels whose changes did (1) differ between groups, and (2) correlate with concurrent behavioral changes in the DBT (Fig. 2). Put another way, we looked for a reproducible pattern of results consistent with the assumption that structural and/or functional changes of the brain during learning mediate the between-group differences in motor learning.

**Figure 3:**
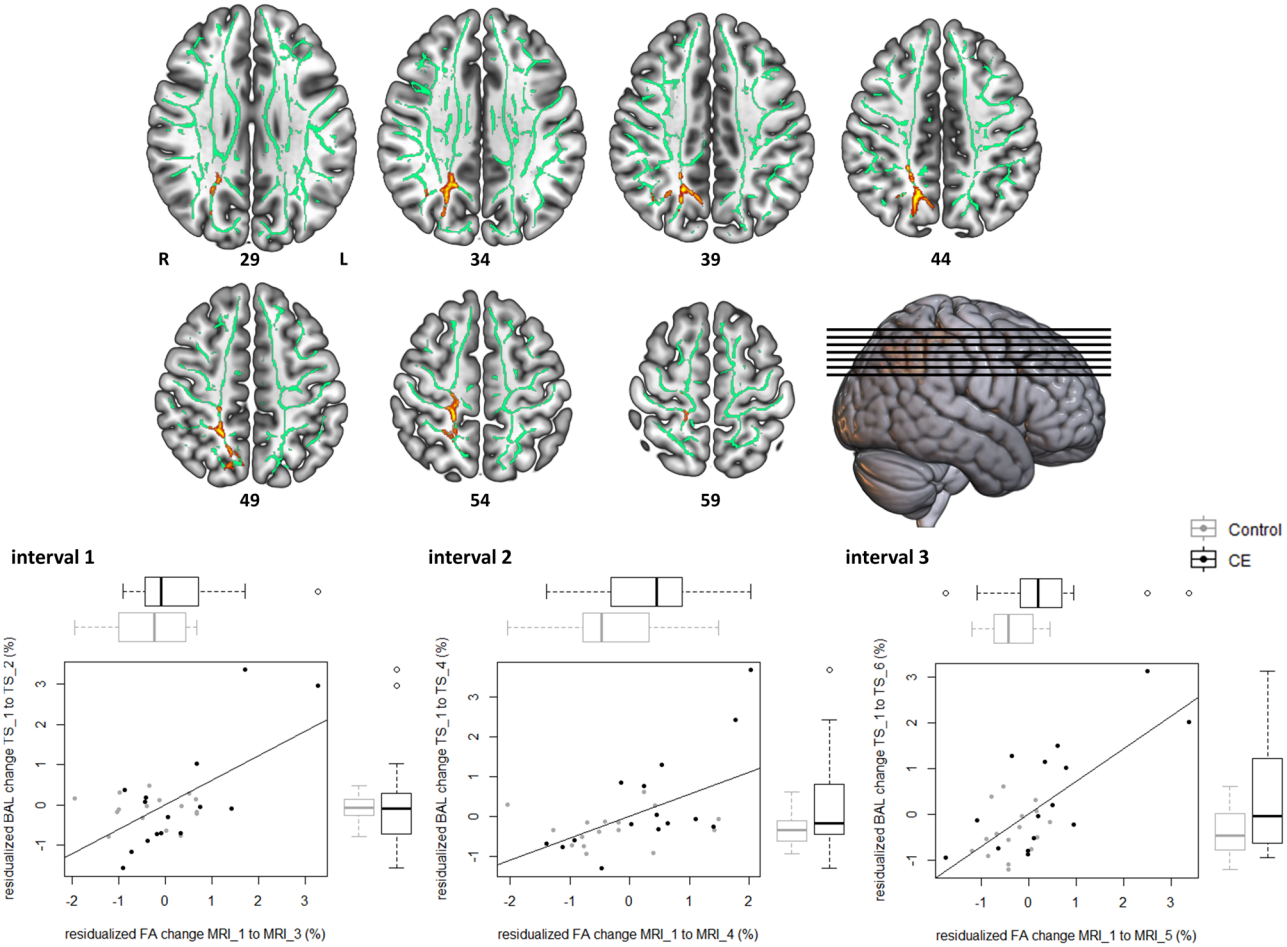
Fractional anisotropy changes (Δ_FA) during three distinct time intervals of learning the DBT covary with treatment (CE vs. control) and concurrent DBT performance changes (time balancing, BAL). Top row: Results from the UIT on baseline-adjusted (residualized) Δ_FA maps based on the NPC methodology. Significant clusters depict voxels, in which UIT revealed evidence for consistent between-group differences regarding Δ_FA (corrected for age and sex) as well as consistent correlations between Δ_FA and (residualized) DBT performance changes (corrected for age, sex and group). Data was visualized using MRIcroGL (https://www.mccauslandcenter.sc.edu/mricrogl/home). Clusters are displayed at *p* < 0.05, FWE-corrected (TFCE) and fattened with the “tbss_fill” script for the purpose of better visualization. Bottom row: Descriptive data illustrating the results of the UIT. For each time interval under examination (cf. Fig. 2), a partial regression scatterplot with line of best fit shows the relation between Δ_FA (within-cluster average in SD units) of the respective time interval and concurrent DBT performance changes from TS_1 (in SD units), corrected for the influence of age and sex. Adjacent boxplots visualize between-group differences in Δ_FA and DBT performance changes, respectively. Note that *z*-scores < 0 indicate subjects whose change scores decreased more than could be linearly predicted from the covariates, and reverse for *z*-scores > 0.

With respect to the diffusion index fractional anisotropy (FA), a measure reflecting the directionality of diffusion in each voxel^52^, NPC analysis revealed significant results (*p*FWE < 0.05) in white matter mainly beneath the parieto-occipital area of the right hemisphere (Fig. 3), including the Precuneous Cortex, superior division of the Lateral Occipital Cortex, Superior Parietal Lobule, and Postcentral Gyrus (Table 1). The observed pattern of results indicates, across all three time intervals under examination, that FA change from baseline was higher in the CE-group than in the control group. FA increases were in turn consistently correlated with concurrent DBT performance improvements, irrespective of group (Fig. 3).

**Table 1:** Peak voxel coordinates and localization of significant clusters emerging from the voxel-based NPC analyses (Figs. 3 and 4).

Cluster Index	Cluster extent	Maximum *p*-value	Peak voxel (MNI152)			Most prominent structures in clusters^53,54^
**Fractional anisotropy (Δ_FA)**						
4	1437	0.018	18	− 63	44	White matter beneath the right parieto-occipital area, including precuneous cortex, superior parietal lobule, superior division of the lateral occipital cortex and postcentral gyrus
3	79	0.04	30	− 62	41	
2	61	0.047	38	− 60	36	
1	14	0.049	24	− 59	45	
**Radial diffusivity (Δ_λ**_**⊥**_**)**						
7	264	0.036	41	− 18	34	White matter beneath the right precentral and postcentral gyri, right superior longitudinal fasciculus
6	87	0.045	39	− 7	30	
5	22	0.049	35	− 19	35	
4	10	0.048	49	1	26	
3	6	0.049	44	− 21	35	
2	2	0.05	49	− 5	20	
1	1	0.05	53	3	26	

**Figure 4:**
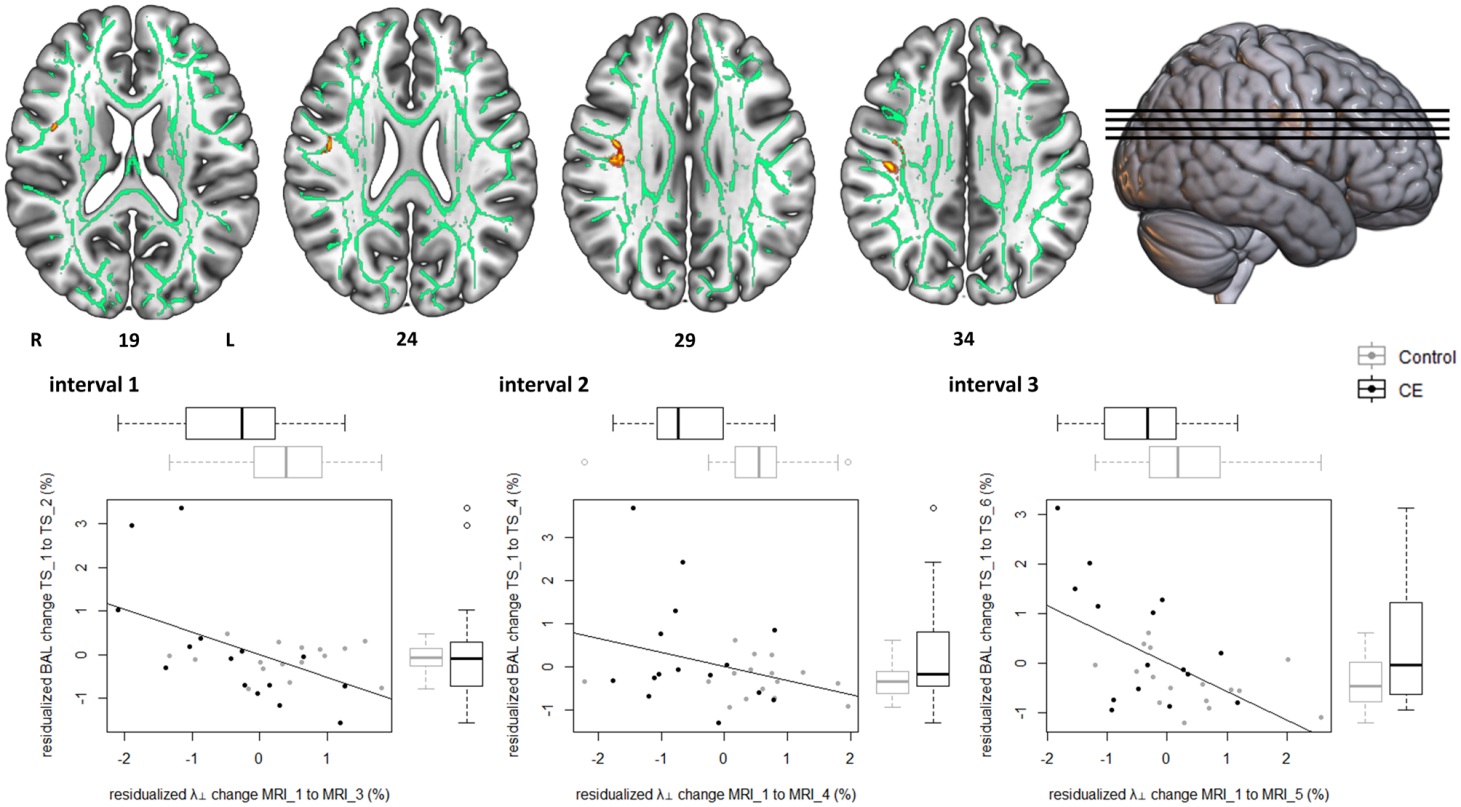
Radial anisotropy changes (Δ_λ_⊥_) during three distinct time intervals of learning the DBT covary with treatment (CE vs. control) and concurrent DBT performance changes (time balancing, BAL). Top row: Results from the UIT on baseline-adjusted (residualized) Δ_λ_⊥_ maps based on the NPC methodology. Significant clusters depict voxels, in which UIT revealed evidence for consistent between-group differences regarding Δ_λ_⊥_ (corrected for age and sex) as well as consistent correlations between Δ_λ_⊥_ and (residualized) DBT performance changes (corrected for age, sex and group). Data was visualized using MRIcroGL (https://www.mccauslandcenter.sc.edu/mricrogl/home). Clusters are displayed at *p* < 0.05, FWE-corrected (TFCE) and fattened with the “tbss_fill” script for the purpose of better visualization. Bottom row: Descriptive data illustrating the results of the UIT. For each time interval under examination (cf. Fig. 2), a partial regression scatterplot with line of best fit shows the relation between Δ_λ_⊥_ (within-cluster average in SD units) of the respective time interval and concurrent DBT performance changes from TS_1 (in SD units), corrected for the influence of age and sex. Adjacent boxplots visualize between-group differences in Δ_λ_⊥_ and DBT performance changes, respectively. Note that *z*-scores < 0 indicate subjects whose change scores decreased more than could be linearly predicted from the covariates, and reverse for *z*-scores > 0.

Likewise, we obtained significant NPC results (*p*FWE < 0.05) for radial diffusivity (λ_⊥_), a diffusion index reflecting water diffusivity perpendicular to axonal fiber tracts, mainly in the right superior longitudinal fasciculus beneath primary motor and somatosensory areas (Fig. 4 and Table 1). Expectedly, in contrast to FA results, results suggest that λ_⊥_ decreased more in the CE than in the control group, along with a negative correlation between λ_⊥_ changes and DBT performance changes (Fig. 4).

**Figure 5:**
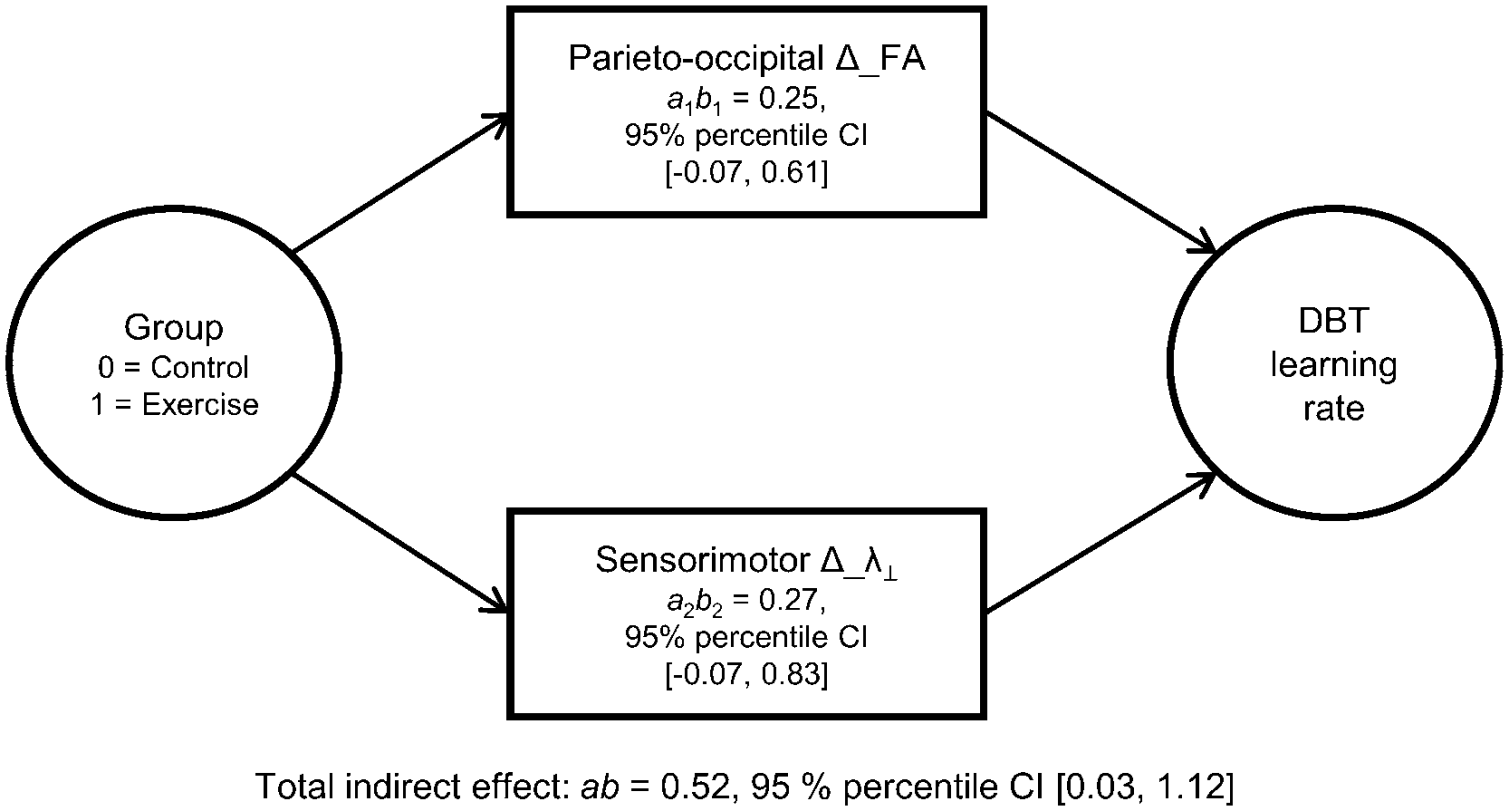
Exercise-induced neuroplasticity conveys the effect of treatment on motor learning. The multiple mediator model shows the relationship between allocation to treatment (Group) and baseline-adjusted (residualized) DBT learning rate, transmitted via residualized white matter changes and corrected for the influence of age and sex. CIs not including zero indicate significant indirect effects.

As a sanity check, we followed up voxel-based NPC results for FA and λ_⊥_ with multiple mediator analysis (Fig. 5). To this end, we averaged residualized change images (intervals 1–3) within-modality and subsequently extracted averaged voxel values within significant clusters. Taken as a set, neuroplastic changes in FA and λ_⊥_ during learning mediated the effect of group (CE vs. controls) on DBT learning rate (*ab* = 0.52, 95% percentile CI [0.03, 1.12], bootstrapped standard error [SE] = 0.28). Thus, the CE group’s DBT learning rate was 0.52 standard deviations higher than the control group’s as the result of FA and λ_⊥_ changes, corrected for the influence of age and sex. Specific indirect effects for both FA (*a*_1_*b*_1_ = 0.25, 95% percentile CI [− 0.07, 0.61], bootstrapped SE = 0.18) and λ_⊥_ (*a*_2_*b*_2_ = 0.27, 95% percentile CI [− 0.07, 0.83], bootstrapped SE = 0.23) clearly trended toward significance.

Further whole-brain analyses on gray matter volume, cerebral blood flow, and network centrality measures based on resting-state fMRI (eigenvector centrality, degree centrality) showed no significant results.

### Time course of white matter plasticity differs between subjects receiving CE and controls

**Figure 6:**
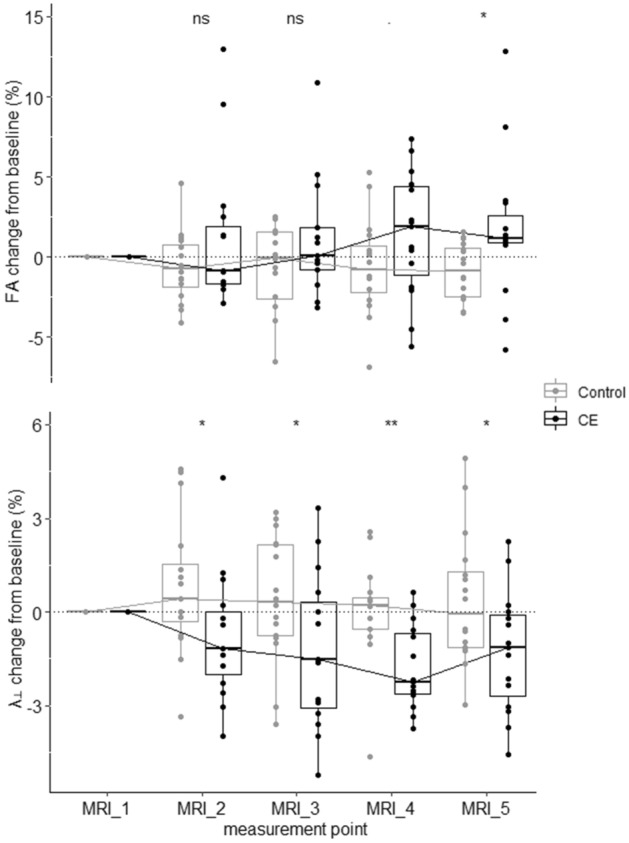
Grouped box chart of indexed FA (top) and λ_⊥_ (bottom) data during the experiment. Indexed data was calculated based on averaged voxel values within significant clusters emerging from the NPC analyses (Figs. 3 and 4). One-sided permutation *p*-values (Table 2) reflecting between-group differences at different measurement points are depicted as follows: ** for *p* ≤ 0.01, * for *p* ≤ 0.05, for *p* ≤ 0.1, ns for *p* > 0.1.

So far, we have demonstrated that learning-related white matter plasticity differs between subjects receiving CE and controls, and that these changes are related to improved DBT learning in the CE group. As a next step, we aimed to disentangle whether white matter changes were immediately present after the CE intervention, whether they developed during the learning phase, or some permutation of the two. To this end, we visualized changes in FA and λ_⊥_ (averaged within significant clusters, respectively) during the experiment as index plot (Fig. 6). Furthermore, like in exploratory whole-brain analyses, we used the NPC framework to jointly analyse change scores relative to baseline.

Fisher’s chi-square combination^55^ of rank-based partial *p*-values yielded significant global *p*-values for both FA and λ_⊥_, respectively (Table 2). Regarding FA, the CE intervention itself did not induce neuroplastic changes in parieto-occipital white matter. Notwithstanding that, between-group differences in FA successively developed during the learning phase, for *p*-values tended to decrease the longer the learning process lasted (Fig. 6 and Table 2). In contrast to FA, we observed that λ_⊥_ changes relative to baseline differed between groups immediately after the CE intervention. This suggests that the relevant neuroplastic adaptations relevant for improved DBT learning were already in place before learning commenced. Of note, these between-group differences in λ_⊥_ were maintained during the learning phase and were strongest at MRI_4 (Fig. 6 and Table 2).

**Table 2:** One-sided permutation *p*-values based on a studentized Wilcoxon rank-sum statistic^56^ of the global null hypothesis that the intervention (CE vs. control) had no effect on FA/λ_⊥_ changes during the experiment (cf. Fig. 6). *p*-values have been adjusted for multiple comparisons using a closed testing procedure (FWE-correction) ^57^. Fisher’s chi-square combining function^55^ was used to summarize evidence over the partial tests (last column). Effect sizes for between-group comparisons at all time intervals are reported as Cliff’s delta (*d*)^58^ with the related 95% CI. The magnitude of *d* can be interpreted using the following thresholds: |*d*|< 0.147 "negligible", |*d*|< 0.33 "small", |*d*|< 0.474 "medium", otherwise "large"^59^.

	MRI_1–MRI_2	MRI_1–MRI_3	MRI_1–MRI_4	MRI_1–MRI_5	NPC
FA	*p* = 0.17, *d* = − 0.2, 95% CI [− 0.57, 0.23]	*p* = 0.16, *d* = − 0.19, 95% CI [− 0.55, 0.23]	*p* = 0.072, *d* = − 0.34, 95% CI [− 0.67, 0.1]	*p* = 0.044, *d* = − 0.49, 95% CI [− 0.78, − 0.03]	*p* = 0.027
λ_⊥_	*p* = 0.015, *d* = 0.48, 95% CI [0.05, 0.75]	*p* = 0.039, *d* = 0.38, 95% CI [− 0.05, 0.69]	*p* = 0.006, *d* = 0.64, 95% CI [0.21, 0.86]	*p* = 0.017, *d* = 0.43, 95% CI [0.01, 0.72]	*p* = 0.004

### Evidence of coupling between structural and functional brain plasticity

Finally, we examined whether white matter plasticity as presented in the previous sections was accompanied by changes in the amplitude of focal spontaneous brain activity (amplitude of low-frequency functional fluctuations, ALFF^60^). ALFF values were extracted for each participant and measurement point in the gray matter adjacent to significant clusters (peak voxels) of the whole-brain NPC analyses (see Table 1). ALFF changes from baseline were then subjected to NPC analyses, which consistently revealed that cortical ALFF decreased more in the CE group compared to controls (Table 3 and Supplementary Information).

**Table 3:** One-sided permutation *p*-values based on a studentized Wilcoxon rank-sum statistic^56^ of the global null hypothesis that the intervention (CE vs. control) had no effect on ALFF changes during the experiment (see Supplementary Information, for indexed box charts). *p*-values have been adjusted for multiple comparisons using a closed testing procedure (FWE-correction)^57^. Fisher’s chi-square combining function^55^ was used to summarize evidence over the partial tests (last column). Effect sizes for between-group comparisons at all time intervals are reported as Cliff’s delta (*d*)^58^ with the related 95% CI. The magnitude of *d* can be interpreted using the following thresholds: |*d*|< 0.147 "negligible", |*d*|< 0.33 "small", |*d*|< 0.474 "medium", otherwise "large"^59^.

	MRI_1–MRI_2	MRI_1–MRI_3	MRI_1–MRI_4	MRI_1–MRI_5	NPC
FA_cluster04 18, − 63, 44	*p* = 0.17, *d* = 0.25, 95% CI [− 0.18, 0.60]	*p* = 0.076, *d* = 0.33, 95% CI [− 0.09, 0.65]	*p* = 0.029, *d* = 0.55, 95% CI [0.12, 0.81]	*p* = 0.29, *d* = 0.13, 95% CI [− 0.31, 0.53]	*p* = 0.017
FA_cluster03 30, − 62, 41	*p* = 0.18, *d* = 0.21, 95% CI [− 0.24, 0.58]	*p* = 0.1, *d* = 0.28, 95% CI [− 0.15, 0.61]	*p* = 0.014, *d* = 0.61, 95% CI [0.20, 0.84]	*p* = 0.093, *d* = 0.3, 95% CI [− 0.14, 0.64]	*p* = 0.009
FA_cluster02 38, − 60, 36	*p* = 0.2, *d* = 0.19, 95% CI [− 0.25, 0.57]	*p* = 0.14, *d* = 0.26, 95% CI [− 0.17, 0.60]	*p* = 0.036, *d* = 0.53, 95% CI [0.11, 0.79]	*p* = 0.086, *d* = 0.33, 95% CI [− 0.10, 0.66]	*p* = 0.021
FA_cluster01 24, − 59, 45	*p* = 0.15, *d* = 0.23, 95% CI [− 0.21, 0.60]	*p* = 0.053, *d* = 0.38, 95% CI [− 0.05, 0.68]	*p* = 0.013, *d* = 0.54, 95% CI [0.11, 0.80]	*p* = 0.2, *d* = 0.19, 95% CI [− 0.24, 0.56]	*p* = 0.004
λ_⊥__cluster07 41, − 18, 34	*p* = 0.027, *d* = 0.46, 95% CI [0.03, 0.74]	*p* = 0.046, *d* = 0.36, 95% CI [− 0.06, 0.67]	*p* = 0.04, *d* = 0.42, 95% CI [− 0.01, 0.72]	*p* = 0.12, *d* = 0.25, 95% CI [− 0.19, 0.61]	*p* = 0.017
λ_⊥__cluster06 39, − 7, 30	*p* = 0.039, *d* = 0.43, 95% CI [− 0.01, 0.73]	*p* = 0.036, *d* = 0.42, 95% CI [0, 0.71]	*p* = 0.027, *d* = 0.48, 95% CI [0.06, 0.76]	*p* = 0.15, *d* = 0.23, 95% CI [− 0.21, 0.60]	*p* = 0.012
λ_⊥__cluster05 35, − 19, 35	*p* = 0.058, *d* = 0.43, 95% CI [− 0.01, 0.73]	*p* = 0.062, *d* = 0.36, 95% CI [− 0.06, 0.67]	*p* = 0.052, *d* = 0.41, 95% CI [− 0.02, 0.71]	*p* = 0.19, *d* = 0.19, 95% CI [− 0.24, 0.56]	*p* = 0.028
λ_⊥__cluster04 49, 1, 26	*p* = 0.037, *d* = 0.43, 95% CI [− 0, 0.73]	*p* = 0.022, *d* = 0.47, 95% CI [0.05, 0.74]	*p* = 0.058, *d* = 0.43, 95% CI [− 0.02, 0.73]	*p* = 0.18, *d* = 0.19, 95% CI [− 0.24, 0.56]	*p* = 0.01
λ_⊥__cluster03 44, − 21, 35	*p* = 0.02, *d* = 0.47, 95% CI [0.04, 0.75]	*p* = 0.046, *d* = 0.36, 95% CI [− 0.06, 0.67]	*p* = 0.045, *d* = 0.43, 95% CI [− 0.01, 0.72]	*p* = 0.13, *d* = 0.25, 95% CI [− 0.19, 0.60]	*p* = 0.016
λ_⊥__cluster02 49, − 5, 20	*p* = 0.047, *d* = 0.4, 95% CI [− 0.04, 0.71]	*p* = 0.019, *d* = 0.48, 95% CI [0.06, 0.75]	*p* = 0.021, *d* = 0.49, 95% CI [0.05, 0.77]	*p* = 0.15, *d* = 0.23, 95% CI [− 0.20, 0.59]	*p* = 0.008
λ_⊥__cluster01 53, 3, 26	*p* = 0.034, *d* = 0.45, 95% CI [0.02, 0.74]	*p* = 0.025, *d* = 0.48, 95% CI [0.06, 0.75]	*p* = 0.056, *d* = 0.44, 95% CI [− 0.01, 0.74]	*p* = 0.19, *d* = 0.18, 95% CI [− 0.24, 0.55]	*p* = 0.009

As revealed by repeated measures correlation^61,62^, λ_⊥_ changes consistently associated with ALFF changes in the surrounding gray matter (0.25 ≤ *r*_rm_ ≤ 0.49). Intermodal associations were less incisive for FA changes, where we found one significant (cluster #2: *r*_rm_ = − 0.28), two marginally significant (cluster #4: *r*_rm_ = − 0.16, cluster #1: *r*_rm_ = − 0.17), and one absent (cluster #3: *r*_rm_ = − 0.02) correlation (see Supplementary Information).

## Discussion

Executing novel whole-body movements poses complex control problems on the motor system^27,63^, thus demanding considerable training efforts to (re-)learn, stabilize and improve such tasks^26^. In recent years, mounting evidence has accumulated that performance and learning of complex movements like the DBT is tightly linked to both initial state^36^ and practice-induced (re-)organization of the brain’s neural circuitry^30,31,35^. In this study, we investigated whether priming CE over two weeks affects the temporal dynamics of brain plasticity during complex motor skill learning. Analyzing structural and functional brain changes during 3 different time intervals of DBT learning using the NPC framework, we found that microstructural changes in white matter relative to the pre-intervention baseline consistently differed between controls and subjects receiving CE. Importantly, these between-group differences in neuroplasticity were also meaningfully related to concomitant DBT performance changes, collectively suggesting a mediating role of white matter plasticity to DBT performance changes. Further analyses revealed that functional base activity in the gray matter spatially adjacent to white matter findings was also altered, thus indicating a link between structural and functional plasticity.

According to neuroscientific theories, the presence of transfer is bound on the logic that trained task and transfer task share some neural commonality, and that this shared substrate changes in response to the trained task^12,19^. For example, we found that increases in FA in the white matter underlying parieto-occipital areas of the right hemisphere consistently correlate with DBT improvement, irrespective of group and regardless which interval of the learning phase is examined. The same applies, with a reversed direction of correlations, to radial diffusivity (λ_⊥_) beneath the primary sensorimotor area of the right hemisphere. Therefore, the evidence we present concurs with the notion that white matter plasticity plays a crucial role in motor learning^29,30,64,65^, and that CE was successful in affecting motor learning-related white matter plasticity.

Parieto-occipital FA findings align with previous evidence in young and elderly subjects showing that learning-related increases in nodal hubness^31^ and local gray matter volume^35^ in the very same area are correlated with DBT performance improvements. Likewise, the clusters we identified are also remarkably close to previously reported FA increases in response to another well-established experimental paradigm of complex motor learning, namely learning to juggle^29^. Since involvement of parieto-occipital brain areas in motor learning has been repeatedly reported in the literature^66–68^, these results point to the probability that priming CE targets a general motor learning-relevant network, thus potentially promoting learning in a task-independent fashion. A follow-up NPC analysis of the temporal dynamics of FA plasticity clearly revealed that between-group differences were not present immediately after CE, but instead gradually developed during learning. One possible explanation for this pattern is an additive or cumulated neuroplastic response if adequate learning opportunities or novel experiences follow after a priming CE intervention^13,69^. For example, past evidence from animal models points to the probability that CE evokes certain learning-relevant molecular and cellular level changes^5,40,44^, which might however not be detectable via the MR imaging signal in humans^13^. Nevertheless, such changes may crucially contribute to a fertile milieu in the brain by laying the foundations for subsequent plasticity and learning^1,5,43^. Alternatively, different time courses of FA plasticity might not be a consequence of priming CE, but simply a consequence of diverging learning curves of the CE and control groups over time (see Ref.^6^). Whether or not the parieto-occipital time course of white matter plasticity is primarily determined by CE or by performance improvements can only be determined if plasticity itself is manipulated, for example by means of an interfering non-invasive brain stimulation protocol.

Compared to parieto-occipital FA, a different pattern of plasticity was observed for λ_⊥_ changes in the white matter beneath primary sensorimotor brain areas. In line with our previous study^6^, we observed that λ_⊥_ in these very regions was already reduced immediately after cessation of CE. This aligns also with other work showing white matter remodelling in primary sensorimotor areas after CE training in young- to middle-aged subjects^70,71^. Interestingly, further analyses in the present paper showed that CE-induced for λ_⊥_ changes are not merely a temporary phenomenon, but that they are persisting during six weeks of motor learning and do not return to the pre-intervention baseline. These results concur well with the idea that CE itself builds up a “structural repertoire” in the white matter, which can then be exploited during motor skill learning^19,36,37^.

Cognitive and motor functions can be viewed as the behavioral outcome of collaborative processing of sensory information by distributed but interconnected neural systems^72^. Against this background, we asked whether changes in structural connectivity as observed by whole-brain NPC analyses were accompanied by changes that are measurable using blood-oxygenation level-dependent (BOLD) signals. Indeed, we found that learning-related changes in local spontaneous neural activity (ALFF) consistently differed between groups. Specifically, we found a greater ALFF reduction relative to the pre-intervention baseline in subjects receiving CE compared to controls. At present, a well-grounded interpretation of ALFF reductions is difficult due to the general lack of studies investigating the effects of CE and/or motor learning on ALFF. It should especially be noted that there is no one-to-one mapping between (local) spontaneous activity and (global) network measures like hubness or centrality^73^, and stronger spontaneous BOLD fluctuations can even be a sign of decreased functional connectivity^74^. Furthermore, it has been shown that local white matter volume as assessed with voxel-based morphometry negatively correlates with ALFF^75^, which is consistent with the intermodal DTI-ALFF correlations that we observed. Therefore, we interpret the reduced power in the low‐frequency band as a positive adaptation to training, for it is a consistent effect across ROIs and time that coincides (and in most cases correlates) with structural plasticity. Of note, previous expert-novice comparisons have shown reduced ALFF in the left superior parietal lobule in badminton players^76^. Likewise, fractional ALFF (fALFF) was reduced in several networks, amongst them the frontoparietal and default mode networks, in Tai Chi Chuan practitioners compared to controls^77^. Decreased ALFF has also been reported as a result of training interventions, for example in patients with early psychosis practicing yoga^78^.

It is plausible that a number of limitations might have influenced the results obtained. To begin with, we acknowledge that the consideration of an active control group would have further strengthened the conclusions regarding CE’s effectiveness in enhancing motor learning. Another possible limiting factor is that our study was powered based on the expected behavioral effect of CE on motor learning^6^. In line with methodological guidelines in the field of neuroplasticity research^79^, we stringently corrected the exploratory whole-brain analyses for multiple comparisons, but this comes at the potential cost that our study might have been under-powered to detect small-to-moderate neuroplastic effects. However, this potential problem was brought into account by focusing on reproducible and behaviorally relevant changes of the brain by using the NPC methodology. Although existing evidence suggests that CE interventions straining the anaerobic-lactic energy system may lead to an augmented neuroplastic response^1,6,25,80^, the debate surrounding the question of “optimal” exercise regimens is still ongoing^1,2,4^. Therefore, we cannot comment on whether using a CE regimen with a different combination of crucial parameters (duration, intensity, frequency, timing etc.) would have led to different outcomes. Furthermore, we cannot rule out that subjects' individual responsiveness to CE or motor learning was affected by inherited factors like certain genetic polymorphisms^81,82^. Related to this, another downside factor regarding our methodology is that we were not able to analyze a potential interaction between sex and the responsiveness to CE due to the too small and unbalanced sample. Finally, we acknowledge that a systematic assessment of sleep behavior^83^ would have further strengthened our conclusions regarding CE’s effectiveness in enhancing motor learning.

Further work needs to be carried out to establish whether our results would generalize to the acquisition of other types of motor skills^26,27^, different stages of the motor skill learning process (i.e., fast vs. slow learning^46^), and to other domains of application like rehabilitation (e.g., in patients suffering from neurological disorders^9–11^). Another interesting opportunity for future research could be to directly compare the effectiveness of different CE intervention strategies on complex motor skill learning, e.g., performing single bouts of CE in close temporal proximity to practice sessions vs. long-term CE interventions prior to the learning phase^1–4^. Current evidence suggests that the mechanisms of action by which acute CE (prior to or after learning) and long-term CE influence motor learning are at least partially different from one another (see Refs.^1–3^, for reviews), which might in turn lead to different effects on learning. Finally, although our results fulfil the statistical requirements of an indirect effect^84^, establishing causality by directly manipulating the assumed central nervous mechanisms of action would certainly be an important contribution to further advance the field^85^.

In this study, we aimed at understanding neural plasticity that governs long-term motor skill learning after a 2-week CE intervention in the intact brain. Our results suggest that improved motor learning following CE is neurobiologically underpinned by altered temporal dynamics of plasticity in task-relevant networks during learning. This lends support to the notion that CE has the potential to affect adaptive neural plasticity in the brain’s motor circuitry^1,2,12,18^. Given that neuroplastic change is considered a basic prerequisite for successful motor skill learning^28–31,64,65^, the findings presented herein might have promising practical implications for different fields of application.

## Materials and methods

### Participants and experimental design

Most experimental procedures including sample size planning were extensively described in our previous paper^6^, such that we will focus on a brief description of the methods in the following. For this randomized controlled trial, a total of 34 healthy, right-handed adults aged 18–35 years (3 dropouts due to illness or injury) were recruited. Exclusion criteria were contraindications to MRI, body mass index (BMI) > 30 kg/cm^2^, a history of neuropsychiatric diseases, left-handedness, self-reported physical activity of > 4 h/week, prior experience with the DBT, and past or present performance-oriented participation in endurance and/or coordinative-demanding sports. The study was performed in accordance with the ethical standards as laid down in the 1964 Declaration of Helsinki and its later amendments. Approval was granted by the Ethics Committee of the University of Leipzig (175-11-30052011) and the study was retrospectively registered in the German Clinical Trial Register (DRKS00025337; date of full registration: 18/05/2021). Written informed consent was obtained from all participants. Prior to participation, all subjects underwent neurological examination as assessed by a credentialed physician.

Subjects were randomly (and gender-balanced) assigned to a CE intervention (*n* = 15) or an inactive control group (*n* = 16; see Ref.^6^ for group characteristics). All participants engaged in six consecutive weeks of learning the DBT^30,31,35^. Before learning commenced, the intervention group underwent a total of seven supervised and individually tailored CE sessions dispersed over two weeks, whereas the control group continued with their habitual activities (life as usual) in parallel (Fig. 1). The rate of adherence was 100% for both CE intervention and DBT learning.

### Cardiovascular fitness assessment

Before engaging in two weeks of either exercise or life-as-usual, all participants performed a graded incremental exercise test (GXT) on a bicycle ergometer (Ergoline ergoselect 200, Bitz, Germany). We used the standard scheme of the World Health Organization (WHO) with an initial work intensity of 25 W and an increase of 25 W every 2 min (pedalling rate 60–70 rpm)^86^. The GXT was terminated after completion of the stage during which a heart rate of 170 bpm was reached. Heart rate was continuously monitored (Polar Elektro Oy H7, Kempele, Finland) and capillary whole blood samples were drawn from a hyperaemic earlobe 15–25 s before the end of each two-minute GXT stage.

Body weight-adjusted power output (physical working capacity, PWC) at fixed heart rates of 120 bpm (PWC120) and 170 bpm (PWC170) was determined by linear interpolation of the workload–heart rate pairs^87^. Lactate concentrations were determined photometrically using a laboratory analyser. Workload–lactate pairs were fitted with a degree three polynomial and two body weight-adjusted indices of cardiovascular fitness were calculated. These were the workload at a fixed lactate concentration of 3 mmol/l (P_3_) and the individual anaerobic threshold (IAT) determined with the “1.5 mmol method” (as described in Ref.^88^). Body weight-adjusted P_3_ and IAT are both recognized as valid indicators of maximal lactate steady state and therefore cardiovascular fitness^89,90^. A GXT post-test was not scheduled since we did not expect fitness gains exceeding a familiarization effect in a training period as short as 2 weeks^6^.

### Cardiovascular exercise intervention

Participants of the CE group performed seven supervised training sessions of cycling spread over 2 weeks. The aim of the CE intervention was to repeatedly expose subjects to exercise-induced hyperlactatemia within the intervention period^1,25^, but without evoking an undesired overtraining/overreaching state^91^ that potentially exerts a negative effect on brain plasticity^92^. The theoretical background is the assumption that lactate produced from active muscles during exercise enters the brain, where it triggers several beneficial neuroplastic responses (see Refs.^1,6,25,80^). Previous research suggests that the lower bound intensity of CE to induce increased brain net lactate uptake is the power or velocity at the “lactate threshold”^93,94^.

Taking these considerations into account, we scheduled an individually tailored exercise protocol with exercise intensity varying between PWC120 and PWC170 (see Ref.^6^, for details). Briefly, each training session started with continuous cycling at PWC120 for 5 min, immediately followed by a 3-min-phase with a gradual increase of exercise intensity in 6 steps of 30 s each, up to the individual’s 100% PWC170. This intensity peak was followed by an another 4-min-phase at PWC120 and another 3-min-phase of stepwise increasing workload up to PWC170. The training session ended after a cooling down phase at PWC120 for 4 min (overall session duration: 19 min). In the second week of training, the total duration of each training session was increased by 2 min by prolonging the time of the two intensity peaks (see Ref.^6^). This adaptation of the CE protocol was done in order to avoid a habituation effect that potentially results in a reduced neuroplastic response^95^.

To test whether the CE intervention was successful in straining the anaerobic lactic metabolism, we drew blood samples from the hyperemic earlobe at regular intervals during one training session of week 1 (19 min program) and one training session of week 2 (21 min program)^6^. The resulting mean lactate value of each participant was subsequently normalized to the individual’s IAT (in terms of the absolute lactate value) and compared against *μ*_*0*_ = 100 by means of a one-sample *t*-test. The average lactate concentration measured during training was significantly higher (mean difference = 44.48%, 95% CI [18.98, 69.98]) than the IAT, *t*(14) = 3.74, *p* = 0.002.

### Whole-body dynamic balancing task (DBT) and quantification of motor learning

After two weeks of CE or control period, subjects engaged in six weeks of DBT training on a seesaw-like platform (stability platform, model 16030, Lafayette Instrument, Lafayette, IN, USA)^96,97^ with one training session (TS) each week^30,31,35^. The platform is moveable in a medio-lateral direction with a maximum deviation of ± 26° on either side. Each training session consisted of 15 trials^30,31,35^ with an inter-trial break of 2 min to avoid fatigue^97^. Standing with both feet on the platform, subjects were instructed to keep the board in a horizontal position for as long as possible during a 30-s trial^30^. The behavioral outcome measure was the time (millisecond timer) in which subjects kept the platform in a horizontal target interval of ± 3° on either side (time balancing, BAL). After each trial subjects received verbal feedback about their BAL (knowledge of results), whereas no feedback regarding strategy or other aspects of the task was provided (discovery learning approach). During task execution, participants' attention was directed to a fixation cross affixed to the wall in front of them (external focus of attention^96^).

Behavioral indices of motor learning as described in the following were calculated after first averaging the 15 BAL values belonging to the respective training session. For exploratory whole-brain NPC analyses, residualized percentage change scores calculated as depicted in Fig. 2 (submodels 4–6) were used as regressors. For statistical mediation analysis (see Fig. 5), we fitted a general power function^98^ to the DBT performance data of each individual, as described in Ref.^6^. We then used the slope value of the power function, adjusted for baseline DBT performance^99^, as dependent variable in the mediation model.

Analysis of behavioral data on motor skill learning as outlined previously focused on motor skill acquisition over 6 weeks, calculated based on averaged within-session motor skill performance^6,46^. Because CE, especially when performed in temporal proximity to motor skill practice, is also thought to affect *online learning* and *motor skill consolidation*^1–4^, two additional statistical analyses were performed.

To address whether CE exerted an influence on the change of performance within single training sessions (online learning^46^), we first fitted a regression line to the BAL data of each training session and each participant. Therefore, we ended up with six slopes and six intercepts per subject. Due to the fact that initial task performance and the rate of subsequent learning are typically negatively correlated^99^, we partialled out the variance associated with the intercept from the slopes. The six resulting residualized slopes were then subjected to an NPC analysis^50^. Specifically, between-group comparisons were conducted on each score using rank-based permutation tests (studentized version of Wilcoxon’s rank sum test^56^) with 1000 permutations. Family-wise error rate of partial *p*-values was adjusted using a closed testing procedure^57^. In addition, we calculated the effect size Cliff’s delta^58^ for each between-group comparison. Note that there is no direct correspondence between the NPC-derived partial *p*-values on the one hand and Cliff’s delta on the other hand since both methods use different ways for calculating differences in the central tendencies between groups. All of the above was done using the NPC v1.1.0^50,100^ and effsize v0.8.0^59^ packages running in R v3.6.1^101^.

To investigate whether CE affected motor skill consolidation, we calculated percent relative retention scores^47^ for all consecutive training sessions (see Fig. 1). We started by averaging the first two trials of a given training session TS_i_ and the last two trials of the immediately preceding training session TS_i-1_, respectively. Note that via averaging we aimed to reduce contamination of retention scores by both continued learning during retention and warm-up decrement^47,48^. Percent relative retention was then computed according to the formula

1 $$Retention\, Score\, of\, {TS}_{i-1} (\%)= \frac{Initial\, Performance\, {TS}_{i}\cdot 100}{Final\, Performance\, {TS}_{i-1}}-100.$$

As described in the previous paragraph, the five resulting retention scores (TS_1–TS_2, TS_2–TS_3, TS_3–TS_4, TS_4–TS_5, TS_5–TS_6) were then subjected to another NPC analysis^50^.

### MR image acquisition

MRI data were acquired on a 3 T MAGNETOM Prisma system (Siemens Healthcare) using a 32-channel head coil. We used the same protocol for each volunteer and each scanning session. The imaging protocol consisted of a series of MRI sequences, as outlined below. Whenever possible, subjects were measured at approximately the same time of day during the study. Subjects were asked to relax, keep their mind free of any thoughts, and to move as little as possible. With respect to the functional image acquisitions, they were additionally instructed to stay awake and alert while keeping their eyes closed^102^. A pillow was placed surrounding the sides and the back of the head to minimize head motion and within- as well as between-subject differences in positioning.

Whole-brain diffusion-weighted images were acquired from 88 axial slices with a spatial resolution of 1.72 × 1.72 × 1.7 mm^3^ (no gap) with a twice-refocused spin echo echo-planar-imaging sequence^103^: TE = 80 ms, TR = 11,000 ms, α = 90°, FOV = 220 × 220 mm^2^, matrix: 128 × 128, phase encoding = A ≫ P, parallel imaging: GRAPPA acceleration factor 2^104^. Sixty isotropically distributed diffusion sensitization directions at *b* = 1000 s/mm^2^ were collected. Additionally, seven datasets without diffusion weighting (*b* = 0 s/mm^2^) were acquired initially and interleaved after each block of 10 diffusion-weighted images as anatomical reference for off-line motion correction. The diffusion MRI sequence lasted ≈ 15 min.

Resting state fMRI scans were acquired using T2*-weighted gradient-echo EPI (GE-EPI) with multiband acceleration, sensitive to BOLD contrast^105,106^. A total of 420 whole-brain volumes were acquired using the following parameters: axial acquisition orientation, phase encoding = A ≫ P, echo spacing = 0.67 ms, voxel size = 2.3 mm isotropic, FOV = 202 × 202 mm^2^, matrix = 88 × 88, 64 slices with 2.3 mm thickness, TE = 30 ms, TR = 1400 ms, α = 69°, partial Fourier factor = 7/8, multiband acceleration factor = 4, acquisition bandwidth = 1775 Hz/Px, interleaved slice order. The total acquisition time for rs-fMRI was ≈ 10 min.

T1-weighted anatomical images to investigate gray matter volume and pulsed arterial spin labeling data to investigate cerebral blood flow were also acquired and processed. Since analyses based on these data did not yield significant results or statistical trends in the present study, they are not discussed further (see Ref.^6^, for details).

### Preprocessing of MR images

All imaging modalities were processed as extensively described in a previously published paper^6^. In the following, we therefore focus on a brief description of the applied diffusion and rs-fMRI preprocessing pipelines.

Diffusion-weighted images were processed using tools provided by the FMRIB Software Library v5.0.9. (https://fsl.fmrib.ox.ac.uk/fsl/fslwiki/FSL)^107^. We started by checking for visual artifacts, followed by skull stripping and motion correction^108^ with appropriate correction of gradient directions^109^. Subsequently, a diffusion tensor^110^ was fitted at each voxel. The diffusion indices fractional anisotropy (FA), mean diffusivity (MD) and radial diffusivity (λ_⊥_) were computed from the eigenvalues of the diffusion tensor with the respective formulas^52^. Subsequent steps followed a reliable^111^ and sensitive^112^ TBSS-based^113^ processing routine, starting with determining an unbiased midspace between the five FA images of each participant^114^. In the next step, the original FA images of each participant were linearly registered to the individual midspace and then averaged to generate an FA template^111,112^. Afterwards, each subject’s FA template was nonlinearly aligned^115^ to every other one to identify the most representative template of the sample (target)^113,116^. After warping each subject’s template to the target, images were registered to MNI152 space (FMRIB58 1 mm template) using affine transformation, and a group-average FA image was thinned and binarized with an FA-value of > 0.2 (skeletonization). The midspace-registered FA, MD, and λ_⊥_ maps of all measurement points were projected onto this skeleton using the warp fields created previously.

The rs-fMRI data were processed using the toolbox fMRIPprep 1.1.3^117^, a Nipype^118^ based toolbox. The pipeline included corrections for motion^108^, slice timing^119^ and susceptibility distortions, followed by intra-subject registration to the respective T1-weighted image and spatial normalization^120^ to the ICBM 152 Nonlinear Asymmetrical Template 2009c^121^. After applying spatial smoothing of 6 mm FWHM, nonaggressive denoising^122^ of images was performed including linear detrending, high-pass filtering, corrections for the global signal in the white matter and the cerebrospinal fluid, and correction for motion-related components as identified by independent component analysis (ICA-AROMA)^123^. Shared variance between the noise components as classified by ICA-AROMA and the other nuisance regressors was removed before denoising was performed^117,124^.

We originally computed voxel-wise measures reflecting nodal hubness based on a graph theoretical approach (degree centrality and eigenvector centrality), which however did not yield significant results. However, to test for a potential coupling of structural and functional plasticity on a local level^125,126^, we additionally quantified ALFF^60^ in a range of 0.01–0.08 Hz using the BRANT toolkit^127^. As recommended in the literature, ALFF maps were computed after full nuisance regression^128^ including high-pass filtering^60,127^. Note that we used ALFF instead of fALFF because the former has shown to be a more reliable measure of focal functional base activity^129^.

### Nonparametric combination (whole-brain analysis)

In the Introduction section of this paper, we hypothesized that subjects receiving CE show a different pattern of brain plasticity during the acquisition of a complex, novel motor skill compared to controls. We furthermore hypothesized that, if it is true that priming CE alters the time course of brain plasticity, this effect should also express itself in terms of behavioral differences in learning^18,19,130^. The complex hypothesis of different time courses of plasticity and their behavioral relevance consists of several testable sub-hypotheses. The NPC framework allows to draw a global conclusion regarding a complex theory by “combining multiple pieces of evidence into a single summary measure of support for the theory”^50^.

Separately for each imaging modality, we started by calculating residualized change images^131,132^ between the baseline MRI scan (MRI_1) and the measurements during learning (MRI_3, MRI_4, MRI_5) using FSL’s tool *fsl_glm* (Fig. 1). We defined MRI_1 (pre-intervention) instead of MRI_2 (post-intervention) as baseline for the calculation of change images for two reasons. First, in case that the subjects’ brains are unchanging during CE (i.e., between MRI_1 and MRI_2), it does not matter which of both measurements is chosen as baseline. Second, assuming that the brain actually changes between MRI_1 and MRI_2, MRI_1 has the advantage to be not susceptible to the occurrence of potential renormalization processes^133^ during the learning phase. Such renormalization processes might give rise to misleading conclusions regarding neuroplastic changes and might lead to spurious correlations between neuroplasticity and changes in behavior.

Next, the global hypothesis of CE’s effects was broken down into a set of six sub-hypotheses (general linear models), each sensitive to the empirical predictions of the theory (see Fig. 2, for a graphical overview). The first three sub-models addressed whether CE-induced structural/functional neuroplasticity was determined by exposure to treatment. This resulted in three ANCOVA-type models regressing residualized neuroplastic change on group assignment (CE vs. control) considering age and sex as covariates of no interest. The remaining three contrasts postulate that structural/functional neuroplasticity during learning correlate with concurrent DBT performance changes, independent of group. Specifically, we used regression-type models to test for a linear relationship between (residualized) DBT performance changes and concurrent neuroplastic changes, adjusted for the influence of age, sex and group. To test our theory, we implemented directional contrasts (one-sided tests) based on the anticipated pattern of results^6^ in all sub-models.

Next, we aimed to identify clusters of voxels showing (a) consistent between-group differences in terms of neuroplasticity as well as (b) common neuroplasticity-performance relationships across groups without the need for prior decisions about where to look (exploratory whole-brain analysis). To this end, we used the Permutation Analysis of Linear Models v. alpha115 (PALM) toolbox^51,134^ to jointly analyze the six statistical sub-models (separately for each imaging modality) using a modified NPC approach. NPC begins with analyzing the sub-models separately using synchronized permutations^51^. Afterwards, at each voxel, combined evidence of test statistics over the six sub-tests was produced using Fisher's combining function^55^ in a way that accounts for the dependence among component tests^49–51^. Therefore, the joint statistic is significant if an aggregate of the partial tests is significant^49–51^.

We ran the NPC with 5000 permutations (within-group sign-flippings) of the data to build up the empirical null distribution from which statistical inference was performed. Clusters were formed using threshold-free cluster enhancement (TFCE^135^) and tested for significance at *p* < 0.05 (cluster-based family-wise error correction). To localize the results in stereotactic space, we used the Harvard–Oxford cortical atlas^54^ and the JHU white-matter tractography atlas^53^ as implemented in FSL.

### Statistical mediation

As a sanity check, we followed up significant results from whole-brain NPC analyses with regression-based statistical mediation, which evaluates the decline in the strength of the relationship between a predictor and an outcome when controlled for the influence of putative mechanisms^84^. We evaluated whether the entire set of putative mediators identified via the NPC analyses would transmit the effect of treatment to DBT learning (total indirect effect) as well as the unique contribution of each mediator while controlling for the influence of the other mediators in the model (specific indirect effect).

First, significant clusters emerging from the whole-brain NPC analyses were used as a mask for averaging and extracting voxel values of residualized change for each participant and time interval within the respective modality (cf. Figs. 1 and 2). Next, the residualized change values for each participant were summed up und divided by three in order to get a single variable reflecting the average neuroplastic change during the learning period. Before inclusion into the statistical mediation model, the presence of an acceptable level of collinearity among putative mediators was checked as described elsewhere^6^.

To determine whether neuroplasticity mediates the relationship between the intervention (binary-coded as: control = 0 and CE = 1) and the baseline-corrected DBT learning rate^6^, we calculated a parallel multiple mediator model with bootstrap confidence interval (CI) estimation as implemented in PROCESS v3.5beta^84^ running in R v3.6.1^101^. Resampling-based estimation of the mediated effect imposes no distributional assumptions and has shown to be applicable even in case of small samples (*n* ≈ 25)^136,137^. To keep variation due to the random resampling process to an absolute minimum, 50,000 bootstrap samples were drawn using the percentile method. A heteroscedasticity-consistent standard error and covariance matrix estimator was used^138^. From each of the bootstrap samples the total and specific indirect effects were computed and sampling distributions were empirically generated. With the distribution, 95% confidence intervals (percentile 95% CI) were determined. A significant mediating effect is assumed if the percentile 95% CI of an indirect effect does not contain zero. Age and sex were added to the multiple mediator models as covariates of no interest.

### Nonparametric combination (follow-up analysis)

For a thorough evaluation of the time course of neuroplasticity during the experiment, we conducted a series of additional analyses. We were especially interested in any brain changes significantly deviating from the pre-intervention baseline to determine in detail whether these changes were present immediately after the CE intervention, whether they emerged during the learning phase, or some permutation of the two.

To this end, within significant clusters that emerged from whole-brain NPC analysis, we first extracted and averaged voxel values of the respective modality for each individual and each measurement point. Based on the extracted data, we then calculated percentage change scores of all time points (MRI_2, MRI_3, MRI_4, MRI_5) relative to baseline (MRI_1) (see Figs. 1 and 2). For each modality, we applied another NPC to the data and compared percentage change scores between groups at all time intervals (MRI_1–MRI_2, MRI_1–MRI_3, MRI_1–MRI_4, MRI_1–MRI_5). Between-group comparisons at each time interval were conducted using rank-based permutation tests^56^ with 1000 permutations (cf. statistical analysis of motor skill consolidation above). Again, we applied FWE-correction to partial *p*-values using a closed testing procedure^57^ and combined the partial *p*-values into a global *p*-value using Fisher’s chi-square combination^55^. The effect size Cliff’s delta^58^ was computed for each between-group comparison. All of the above was done using the NPC v1.1.0^50,100^ and effsize v0.8.0^59^ packages running in R v3.6.1^101^.

We were also interested whether learning-related white matter changes were paralleled by changes in resting state functional connectivity. To address this, we created a binary brain mask (sphere with 10 mm radius) around all peak voxels that emerged from significant clusters of the whole-brain NPC analyses (Table 1). Within-sphere ALFF values were then extracted and averaged for each participant and measurement point, but only in voxels where the sphere intersected with the surrounding gray matter (GM threshold ≥ 0.2). We then calculated percentage change scores of ALFF and subjected these to NPC analysis, exactly as described above. Finally, we tested for a statistical intermodal relationship between changes in white matter microstructure and concomitant changes in ALFF by means of a repeated measures correlation^61,62^, where each of the four intervals was treated as a subject with 31 observations. This corresponds to the idea of pooling evidence across bivariate correlations between diffusion and ALFF changes at four time intervals (MRI_1–MRI_2, MRI_1–MRI_3, MRI_1–MRI_4, MRI_1–MRI_5). Repeated measures correlations were conducted using R’s^101^ rmcorr v0.4.3 package^61,139^.

## Supplementary Information

Dataset S1.

Supplementary Information.
