# Supplementary material for: Priming cardiovascular exercise improves complex motor skill learning by affecting the trajectory of learning-related brain plasticity
Source: Sci Rep. 2022 Jan 21;12:1107. doi: 10.1038/s41598-022-05145-7 (PMC8783021; doi:10.1038/s41598-022-05145-7)
Supplement: Supplementary file 2 — Supplementary Information. [file 41598_2022_5145_MOESM2_ESM.docx]

**Priming cardiovascular exercise improves complex motor skill learning by affecting the trajectory of learning-related brain plasticity**

Nico Lehmann, Arno Villringer, & Marco Taubert

**Supplemental Material**

Analysis of motor skill consolidation

Supplementary Table 1: Two-sided permutation p-values based on a studentized Wilcoxon rank-sum statistic [1] of the global null hypothesis that the intervention (CE vs. control) had no effect on motor skill consolidation. P-values have been adjusted for multiple comparisons using a closed testing procedure (FWE-correction) [2]. Descriptive statistics for each group are reported as median and interquartile range (IQR). Effect sizes for between-group comparisons at all measurement points are reported as Cliff’s delta (d) [4] with the related 95% CI. The magnitude of d can be interpreted using the following thresholds: |d| < 0.147 "negligible", |d| < 0.33 "small", |d| < 0.474 "medium", otherwise "large" [5].

|  | relative retention control group (%) | relative retention exercise group (%) | NPC *p*-value (FWE-corr.) and effect size *d* |
| --- | --- | --- | --- |
| TS_1-TS_2 | -17.50  (-32.17, -8.88) | -25.21  (-32.94, -8.77) | *p =* 1, *d* = 0.03, 95% CI [-0.38, 0.42] |
| TS_2-TS_3 | -20.69  (-27.35, -17.97) | -13.01  (-27.06, 3.23) | *p =* .95, *d* = -0.22, 95% CI [-0.58, 0.22] |
| TS_3-TS_4 | -18.42  (-28.00, -7.31) | -13.83  (-21.37, 4.49) | *p =* .97, *d* = -0.18, 95% CI [-0.55, 0.25] |
| TS_4-TS_5 | -20.94  (-29.53, 3.37) | -10.18  (-27.18, 0.65) | *p =* 1, *d* = -0.03, 95% CI [-0.43, 0.39] |
| TS_5-TS_6 | -10.63  (-22.11, 13.62) | -7.01  (-19.37, 3.65) | *p =* 1, *d* = 0.01, 95% CI [-0.30, 0.41] |

Analysis of online learning

Supplementary Table 2: Two-sided permutation p-values based on a studentized Wilcoxon rank-sum statistic [1] of the global null hypothesis that the intervention (CE vs. control) had no effect on online learning. The dependent variable is an adjusted slope representing within-session performance change corrected for the intercept (i.e., estimated performance [BAL in s] of the first trial in a given training session). P-values have been adjusted for multiple comparisons using a closed testing procedure (FWE-correction) [2]. Descriptive statistics for each group are reported as median and interquartile range (IQR). Effect sizes for between-group comparisons at all measurement points are reported as Cliff’s delta (d) [4] with the related 95% CI. The magnitude of d can be interpreted using the following thresholds: |d| < 0.147 "negligible", |d| < 0.33 "small", |d| < 0.474 "medium", otherwise "large" [5].

|  | corrected slope control group | corrected slope exercise group | NPC *p*-value (FWE-corr.) and effect size *d* |
| --- | --- | --- | --- |
| TS_1 | 0.09  (-0.004, 0.25) | 0.06  (-0.11, 0.17) | *p =* .69, *d* = 0.2, 95% CI [-0.23, 0.56] |
| TS_2 | -0.01  (-0.08, 0.07) | -0.01  (-0.13, 0.15) | *p =* .94, *d* = -0.017, 95% CI [-0.42, 0.40] |
| TS_3 | -0.07  (-0.12, 0.06) | -0.02  (-0.09, 0.18) | *p =* .65, *d* = -0.22, 95% CI [-0.58, 0.22] |
| TS_4 | -0.07  (-0.17, 0) | -0.03  (-0.19, 0.18) | *p =* .68, *d* = -0.21, 95% CI [-0.58, 0.24] |
| TS_5 | -0.13  (-0.16, 0.04) | -0.005  (-0.15, 0.25) | *p =* .38, *d* = -0.33, 95% CI [-0.66, 0.11] |
| TS_6 | -0.02  (-0.14, 0.05) | -0.16  (-0.20, -0.04) | *p =* .46, *d* = 0.29, 95% CI [-0.15, 0.63] |

ALFF changes in fractional anisotropy (FA) cluster #04 (Precuneous Cortex/ Lateral Occipital Cortex, superior division) from whole-brain NPC


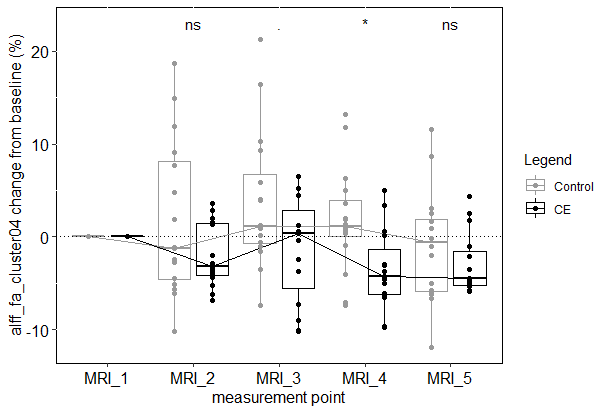


Supplementary Figure 1: Grouped box chart of indexed ALFF data during the experiment. Indexed data was calculated based on averaged, gray matter-only ALFF voxel values within a 10mm sphere centered around 18, -63, 44 (peak voxel of FA cluster #4 from whole-brain NPC analysis). One-sided permutation p-values (see main manuscript, Table 4) reflecting between-group differences at different measurement points are depicted as follows: * for p ≤ 0.05, . for p ≤ 0.1, ns for p > 0.1.


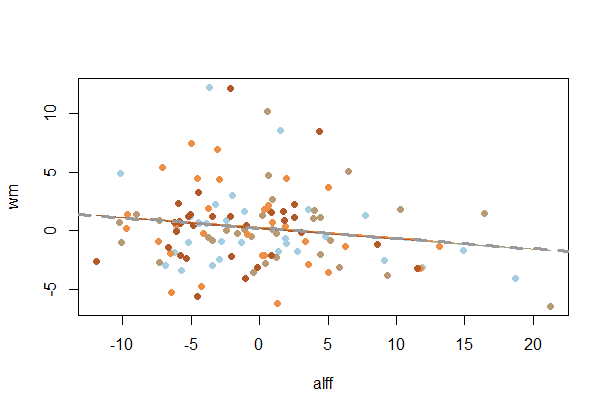


Supplementary Figure 2: Repeated measures intermodal correlation [6,7] between indexed FA changes within FA cluster #4 (see main manuscript, Figure 3, and Table 2) and indexed ALFF changes in the adjacent gray matter (extracted within a 10mm sphere centered around 18, -63, 44), r_rm_(119) = -0.16, 95% bootstrap CI [-0.33, 0.01]). Abscissa and ordinate units are percentage changes relative to MRI_1.

Light blue: interval 1 (MRI_1-MRI_2), beige: interval 2 (MRI_1-MRI_3), orange: interval 3 (MRI_1-MRI_4), brown: interval 4 (MRI_1-MRI_5)

ALFF changes in fractional anisotropy (FA) cluster #03 (Lateral Occipital Cortex, superior division) from whole-brain NPC


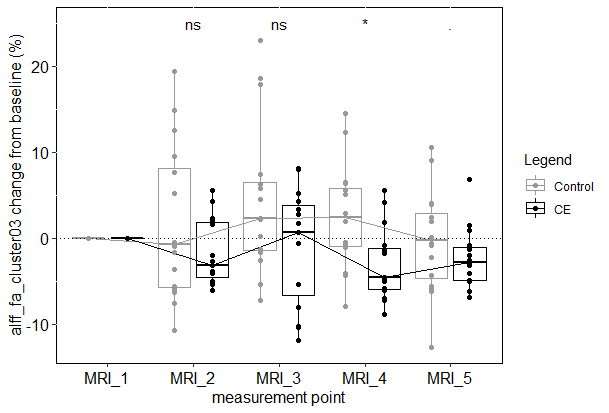


Supplementary Figure 3: Grouped box chart of indexed ALFF data during the experiment. Indexed data was calculated based on averaged, gray matter-only ALFF voxel values within a 10mm sphere centered around 30, -62, 41 (peak voxel of FA cluster #3 from whole-brain NPC analysis). One-sided permutation p-values (see main manuscript, Table 4) reflecting between-group differences at different measurement points are depicted as follows: * for p ≤ 0.05, . for p ≤ 0.1, ns for p > 0.1.


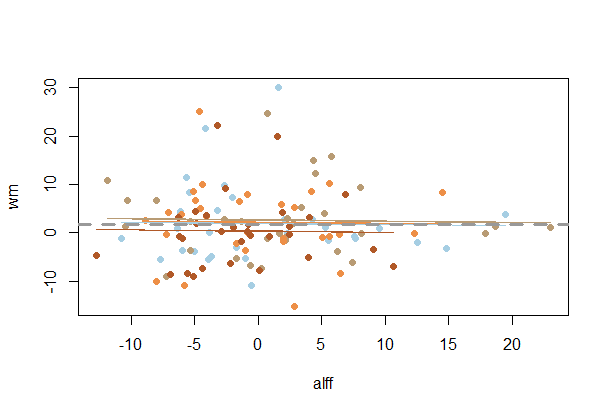


Supplementary Figure 4: Repeated measures intermodal correlation [6,7] between indexed FA changes within FA cluster #3 (see main manuscript, Figure 3, and Table 2) and indexed ALFF changes in the adjacent gray matter (extracted within a 10mm sphere centered around 30, -62, 41), r_rm_(119) = -0.02, 95% bootstrap CI [-0.15, 0.11]). Abscissa and ordinate units are percentage changes relative to MRI_1.

Light blue: interval 1 (MRI_1-MRI_2), beige: interval 2 (MRI_1-MRI_3), orange: interval 3 (MRI_1-MRI_4), brown: interval 4 (MRI_1-MRI_5)

ALFF changes in fractional anisotropy (FA) cluster #02 (Lateral Occipital Cortex, superior division/ angular gyrus) from whole-brain NPC


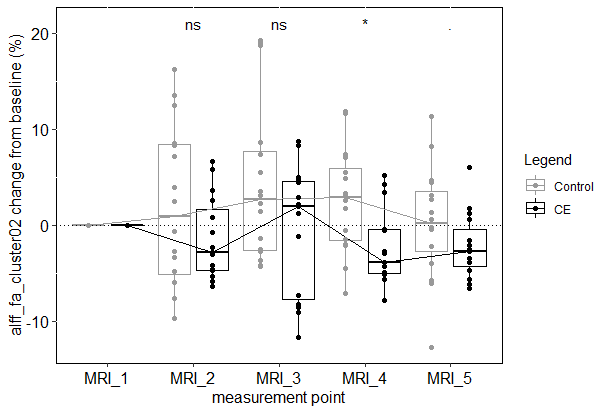


Supplementary Figure 5: Grouped box chart of indexed ALFF data during the experiment. Indexed data was calculated based on averaged, gray matter-only ALFF voxel values within a 10mm sphere centered around 38, -60, 36 (peak voxel of FA cluster #2 from whole-brain NPC analysis). One-sided permutation p-values (see main manuscript, Table 4) reflecting between-group differences at different measurement points are depicted as follows: * for p ≤ 0.05, . for p ≤ 0.1, ns for p > 0.1.


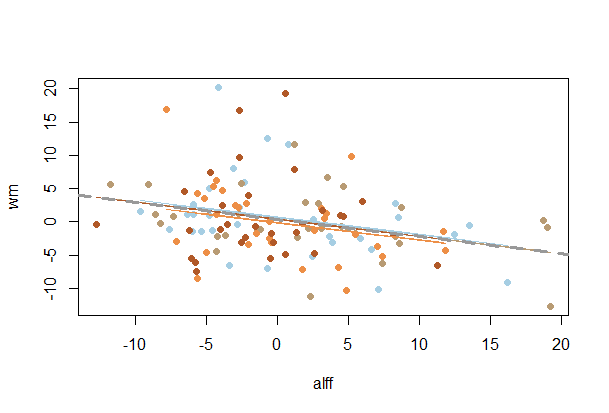


Supplementary Figure 6: Repeated measures intermodal correlation [6,7] between indexed FA changes within FA cluster #2 (see main manuscript, Figure 3, and Table 2) and indexed ALFF changes in the adjacent gray matter (extracted within a 10mm sphere centered around 38, -60, 36), r_rm_(119) = -0.28, 95% bootstrap CI [-0.42, -0.14]). Abscissa and ordinate units are percentage changes relative to MRI_1.

Light blue: interval 1 (MRI_1-MRI_2), beige: interval 2 (MRI_1-MRI_3), orange: interval 3 (MRI_1-MRI_4), brown: interval 4 (MRI_1-MRI_5)

ALFF changes in fractional anisotropy (FA) cluster #01 (Lateral Occipital Cortex, superior division/ Superior Parietal Lobule) from whole-brain NPC


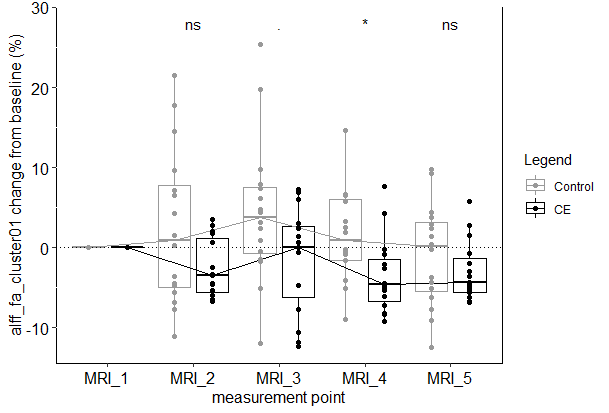


Supplementary Figure 7: Grouped box chart of indexed ALFF data during the experiment. Indexed data was calculated based on averaged, gray matter-only ALFF voxel values within a 10mm sphere centered around 24, -59, 45 (peak voxel of FA cluster #1 from whole-brain NPC analysis). One-sided permutation p-values (see main manuscript, Table 4) reflecting between-group differences at different measurement points are depicted as follows: * for p ≤ 0.05, . for p ≤ 0.1, ns for p > 0.1.


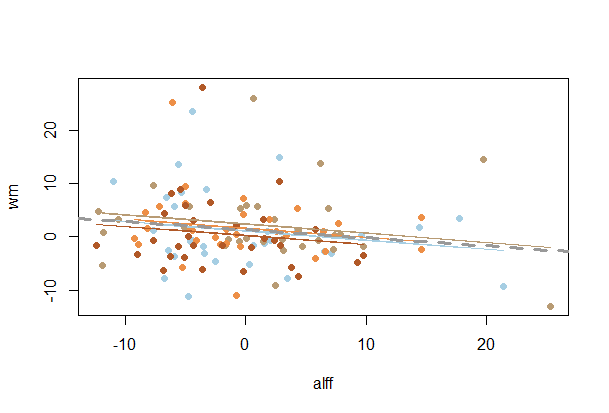


Supplementary Figure 8: Repeated measures intermodal correlation [6,7] between indexed FA changes within FA cluster #1 (see main manuscript, Figure 3, and Table 2) and indexed ALFF changes in the adjacent gray matter (extracted within a 10mm sphere centered around 24, -59, 45), r_rm_(119) = -0.17, 95% bootstrap CI [-0.35, 0.02]). Abscissa and ordinate units are percentage changes relative to MRI_1.

Light blue: interval 1 (MRI_1-MRI_2), beige: interval 2 (MRI_1-MRI_3), orange: interval 3 (MRI_1-MRI_4), brown: interval 4 (MRI_1-MRI_5)

ALFF changes in radial diffusivity (λ_⊥_) cluster #07 (Postcentral Gyrus/ Precentral Gyrus) from whole-brain NPC


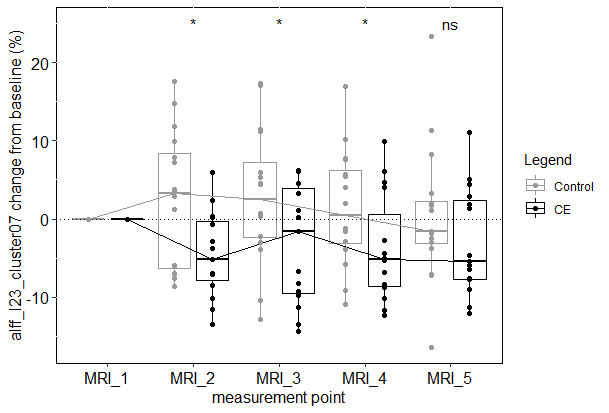


Supplementary Figure 9: Grouped box chart of indexed ALFF data during the experiment. Indexed data was calculated based on averaged, gray matter-only ALFF voxel values within a 10mm sphere centered around 41, -18, 34 (peak voxel of λ_⊥_ cluster #7 from whole-brain NPC analysis). One-sided permutation p-values (see main manuscript, Table 4) reflecting between-group differences at different measurement points are depicted as follows: * for p ≤ 0.05, . for p ≤ 0.1, ns for p > 0.1.


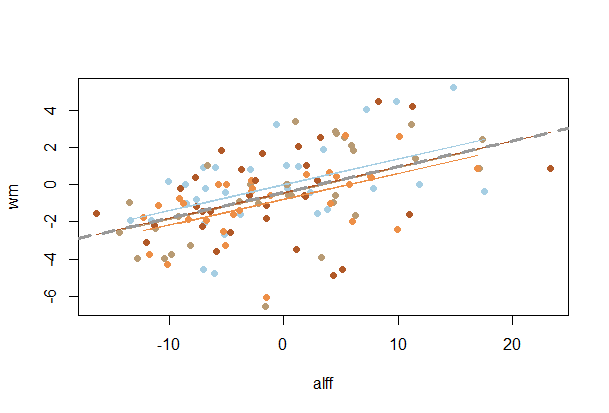


Supplementary Figure 10: Repeated measures intermodal correlation [6,7] between indexed λ_⊥_ changes within λ_⊥_ cluster #7 (see main manuscript, Figure 4, and Table 2) and indexed ALFF changes in the adjacent gray matter (extracted within a 10mm sphere centered around 41, -18, 34), r_rm_(119) = 0.49, 95% bootstrap CI [0.37, 0.62]). Abscissa and ordinate units are percentage changes relative to MRI_1.

Light blue: interval 1 (MRI_1-MRI_2), beige: interval 2 (MRI_1-MRI_3), orange: interval 3 (MRI_1-MRI_4), brown: interval 4 (MRI_1-MRI_5)

ALFF changes in radial diffusivity (λ_⊥_) cluster #06 (Precentral Gyrus/ Postcentral Gyrus) from whole-brain NPC


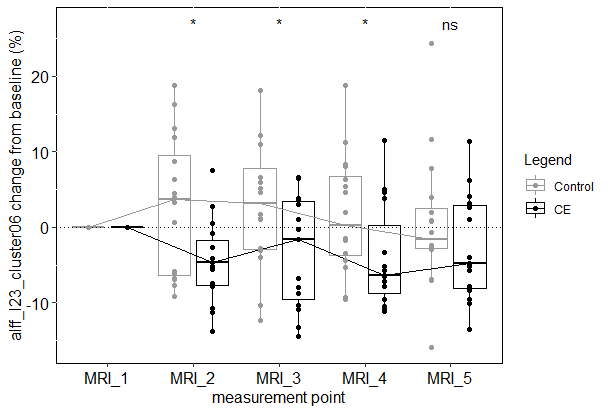


Supplementary Figure 11: Grouped box chart of indexed ALFF data during the experiment. Indexed data was calculated based on averaged, gray matter-only ALFF voxel values within a 10mm sphere centered around 39, -7, 30 (peak voxel of λ_⊥_ cluster #6 from whole-brain NPC analysis). One-sided permutation p-values (see main manuscript, Table 4) reflecting between-group differences at different measurement points are depicted as follows: * for p ≤ 0.05, . for p ≤ 0.1, ns for p > 0.1.


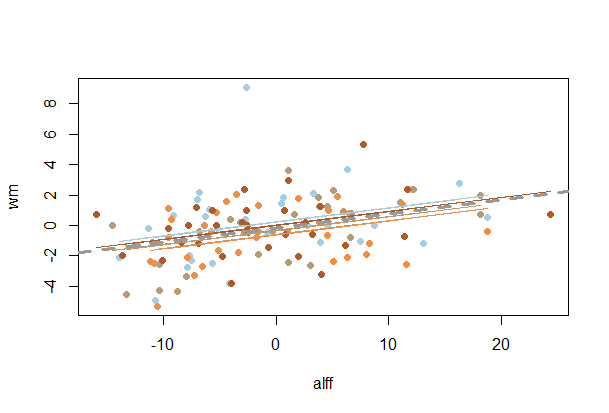


Supplementary Figure 12: Repeated measures intermodal correlation [6,7] between indexed λ_⊥_ changes within λ_⊥_ cluster #6 (see main manuscript, Figure 4, and Table 2) and indexed ALFF changes in the adjacent gray matter (extracted within a 10mm sphere centered around 39, -7, 30), r_rm_(119) = 0.36, 95% bootstrap CI [0.22, 0.51]). Abscissa and ordinate units are percentage changes relative to MRI_1.

Light blue: interval 1 (MRI_1-MRI_2), beige: interval 2 (MRI_1-MRI_3), orange: interval 3 (MRI_1-MRI_4), brown: interval 4 (MRI_1-MRI_5)

ALFF changes in radial diffusivity (λ_⊥_) cluster #05 (Precentral Gyrus/ Postcentral Gyrus) from whole-brain NPC


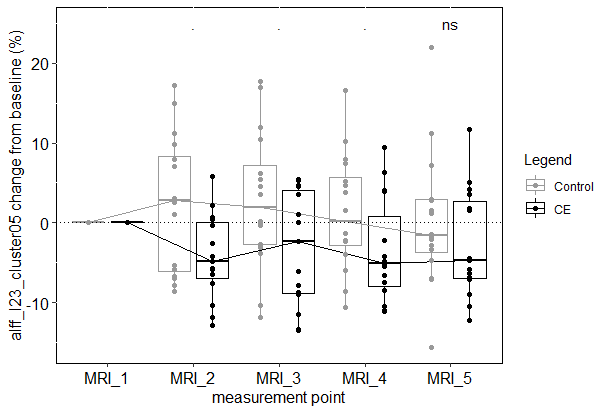


Supplementary Figure 13: Grouped box chart of indexed ALFF data during the experiment. Indexed data was calculated based on averaged, gray matter-only ALFF voxel values within a 10mm sphere centered around 35, -19, 35 (peak voxel of λ_⊥_ cluster #5 from whole-brain NPC analysis). One-sided permutation p-values (see main manuscript, Table 4) reflecting between-group differences at different measurement points are depicted as follows: * for p ≤ 0.05, . for p ≤ 0.1, ns for p > 0.1.


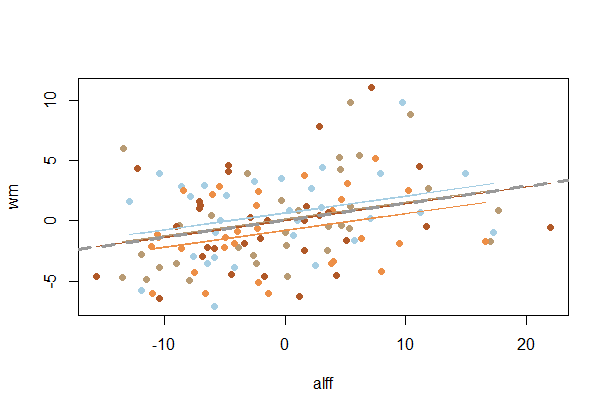


Supplementary Figure 14: Repeated measures intermodal correlation [6,7] between indexed λ_⊥_ changes within λ_⊥_ cluster #5 (see main manuscript, Figure 4, and Table 2) and indexed ALFF changes in the adjacent gray matter (extracted within a 10mm sphere centered around 35, -19, 35), r_rm_(119) = 0.29, 95% bootstrap CI [0.14, 0.45]). Abscissa and ordinate units are percentage changes relative to MRI_1.

Light blue: interval 1 (MRI_1-MRI_2), beige: interval 2 (MRI_1-MRI_3), orange: interval 3 (MRI_1-MRI_4), brown: interval 4 (MRI_1-MRI_5)

ALFF changes in radial diffusivity (λ_⊥_) cluster #04 (Precentral Gyrus/ Inferior Frontal Gyrus, pars opercularis) from whole-brain NPC


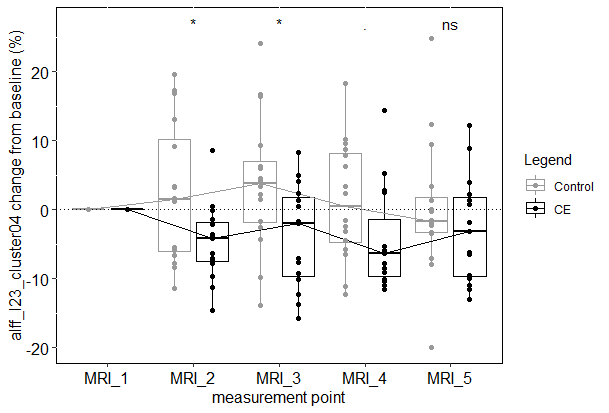


Supplementary Figure 15: Grouped box chart of indexed ALFF data during the experiment. Indexed data was calculated based on averaged, gray matter-only ALFF voxel values within a 10mm sphere centered around 49, 1, 26 (peak voxel of λ_⊥_ cluster #4 from whole-brain NPC analysis). One-sided permutation p-values (see main manuscript, Table 4) reflecting between-group differences at different measurement points are depicted as follows: * for p ≤ 0.05, . for p ≤ 0.1, ns for p > 0.1.


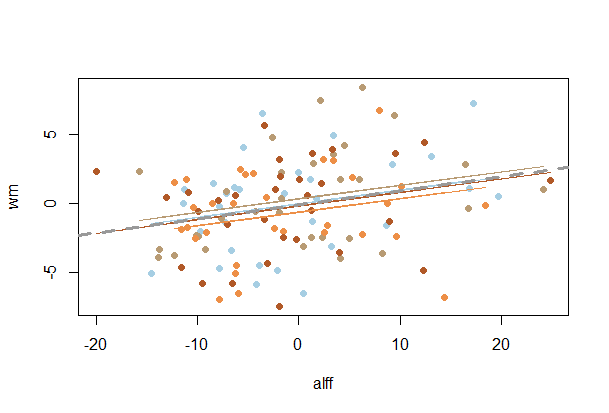


Supplementary Figure 16: Repeated measures intermodal correlation [6,7] between indexed λ_⊥_ changes within λ_⊥_ cluster #4 (see main manuscript, Figure 4, and Table 2) and indexed ALFF changes in the adjacent gray matter (extracted within a 10mm sphere centered around 49, 1, 26), r_rm_(119) = 0.26, 95% bootstrap CI [0.1, 0.4]). Abscissa and ordinate units are percentage changes relative to MRI_1.

Light blue: interval 1 (MRI_1-MRI_2), beige: interval 2 (MRI_1-MRI_3), orange: interval 3 (MRI_1-MRI_4), brown: interval 4 (MRI_1-MRI_5)

ALFF changes in radial diffusivity (λ_⊥_) cluster #03 (Postcentral Gyrus/ Precentral Gyrus) from whole-brain NPC


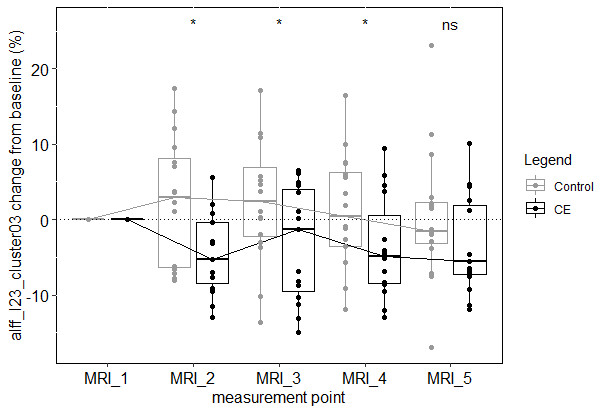


Supplementary Figure 17: Grouped box chart of indexed ALFF data during the experiment. Indexed data was calculated based on averaged, gray matter-only ALFF voxel values within a 10mm sphere centered around 44, -21, 35 (peak voxel of λ_⊥_ cluster #3 from whole-brain NPC analysis). One-sided permutation p-values (see main manuscript, Table 4) reflecting between-group differences at different measurement points are depicted as follows: * for p ≤ 0.05, . for p ≤ 0.1, ns for p > 0.1.


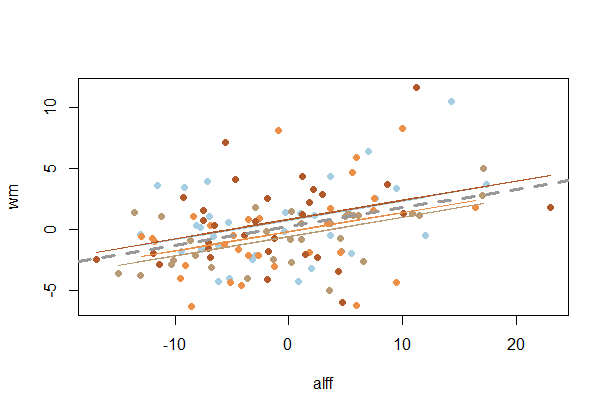


Supplementary Figure 18: Repeated measures intermodal correlation [6,7] between indexed λ_⊥_ changes within λ_⊥_ cluster #3 (see main manuscript, Figure 4, and Table 2) and indexed ALFF changes in the adjacent gray matter (extracted within a 10mm sphere centered around 44, -21, 35), r_rm_(119) = 0.39, 95% bootstrap CI [0.23, 0.53]). Abscissa and ordinate units are percentage changes relative to MRI_1.

Light blue: interval 1 (MRI_1-MRI_2), beige: interval 2 (MRI_1-MRI_3), orange: interval 3 (MRI_1-MRI_4), brown: interval 4 (MRI_1-MRI_5)

ALFF changes in radial diffusivity (λ_⊥_) cluster #02 (Central Opercular Cortex/ Postcentral Gyrus/ Precentral Gyrus) from whole-brain NPC


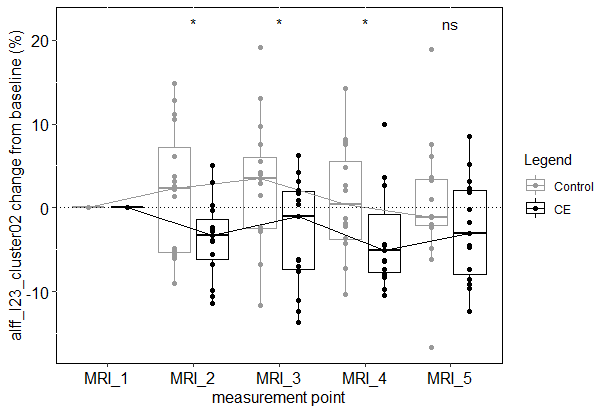


Supplementary Figure 19: Grouped box chart of indexed ALFF data during the experiment. Indexed data was calculated based on averaged, gray matter-only ALFF voxel values within a 10mm sphere centered around 49, -5, 20 (peak voxel of λ_⊥_ cluster #2 from whole-brain NPC analysis). One-sided permutation p-values (see main manuscript, Table 4) reflecting between-group differences at different measurement points are depicted as follows: * for p ≤ 0.05, . for p ≤ 0.1, ns for p > 0.1.


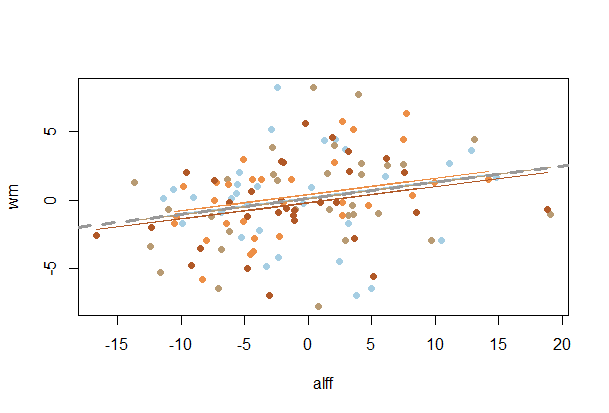


Supplementary Figure 20: Repeated measures intermodal correlation [6,7] between indexed λ_⊥_ changes within λ_⊥_ cluster #2 (see main manuscript, Figure 4, and Table 2) and indexed ALFF changes in the adjacent gray matter (extracted within a 10mm sphere centered around 49, -5, 20), r_rm_(119) = 0.25, 95% bootstrap CI [0.12, 0.4]). Abscissa and ordinate units are percentage changes relative to MRI_1.

Light blue: interval 1 (MRI_1-MRI_2), beige: interval 2 (MRI_1-MRI_3), orange: interval 3 (MRI_1-MRI_4), brown: interval 4 (MRI_1-MRI_5)

ALFF changes in radial diffusivity (λ_⊥_) cluster #01 (Precentral Gyrus/ Inferior Frontal Gyrus, pars opercularis) from whole-brain NPC


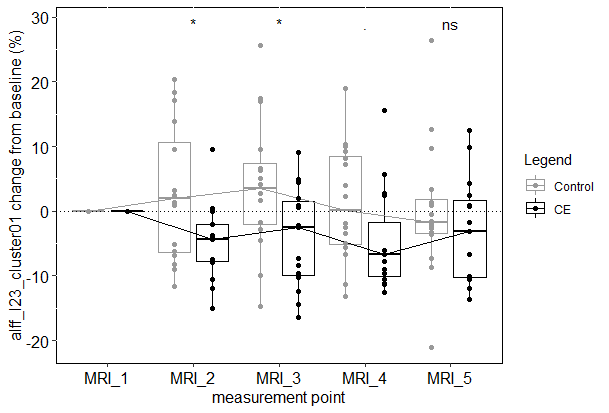


Supplementary Figure 21: Grouped box chart of indexed ALFF data during the experiment. Indexed data was calculated based on averaged, gray matter-only ALFF voxel values within a 10mm sphere centered around 53, 3, 26 (peak voxel of λ_⊥_ cluster #1 from whole-brain NPC analysis). One-sided permutation p-values (see main manuscript, Table 4) reflecting between-group differences at different measurement points are depicted as follows: * for p ≤ 0.05, . for p ≤ 0.1, ns for p > 0.1.


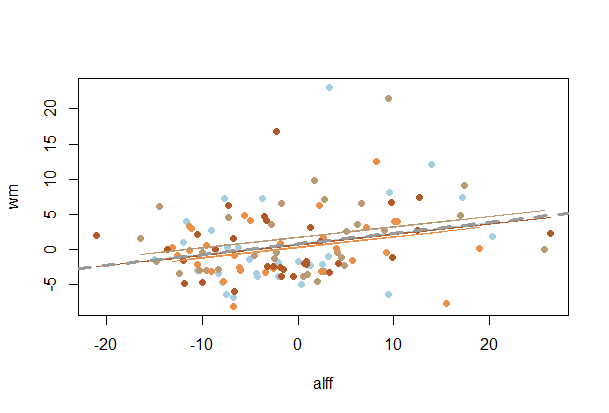


Supplementary Figure 22: Repeated measures intermodal correlation [6,7] between indexed λ_⊥_ changes within λ_⊥_ cluster #1 (see main manuscript, Figure 4, and Table 2) and indexed ALFF changes in the adjacent gray matter (extracted within a 10mm sphere centered around 53, 3, 26), r_rm_(119) = 0.27, 95% bootstrap CI [0.11, 0.4]). Abscissa and ordinate units are percentage changes relative to MRI_1.

Light blue: interval 1 (MRI_1-MRI_2), beige: interval 2 (MRI_1-MRI_3), orange: interval 3 (MRI_1-MRI_4), brown: interval 4 (MRI_1-MRI_5)

References

1. Chung, E. & Romano, J. P. Exact and asymptotically robust permutation tests. *Ann. Statist.* **41,** 484–507 (2013).

2. Marcus, R., Peritz, E. & Gabriel, K. R. On closed testing procedures with special reference to ordered analysis of variance. *Biometrika* **63,** 655–660 (1976).

3. Fisher, R. A. *Statistical Methods for Research Workers.* 4^th^ ed. (Oliver & Boyd, 1932).

4. Cliff, N. *Ordinal methods for behavioral data analysis* (Erlbaum, 1996).

5. Torchiano, M. *Effsize - A Package For Efficient Effect Size Computation v0.8.0* (2016).

6. Bland, J. M. & Altman, D. G. Calculating correlation coefficients with repeated observations: Part 1—Correlation within subjects. *BMJ* **310,** 446 (1995).

7. Bakdash, J. Z. & Marusich, L. R. Repeated Measures Correlation. *Front. Psychol.* **8,** 456 (2017).
